# Effect of traffic volumes on polycyclic aromatic hydrocarbons of particulate matter: A comparative study from urban and rural areas in Malaysia

**DOI:** 10.1371/journal.pone.0315439

**Published:** 2024-12-12

**Authors:** Samer Al-Battawi, Mohd Talib Latif, Vivien How, Karuppiah Thilakavathy, Haris Hafizal Abd Hamid, Chung Keat Tan, Yu Bin Ho

**Affiliations:** 1 Department of Environmental and Occupational Health, Faculty of Medicine and Health Sciences, Universiti Putra Malaysia, Serdang, Selangor, Malaysia; 2 Department of Earth Sciences and Environment, Faculty of Science and Technology, Universiti Kebangsaan Malaysia, Bangi, Selangor, Malaysia; 3 Department of Biomedical Science, Faculty of Medicine and Health Sciences, Universiti Putra Malaysia, Serdang, Selangor, Malaysia; 4 Genetics and Regenerative Medicine Research Group, Faculty of Medicine and Health Sciences, Universiti Putra Malaysia, Serdang, Selangor, Malaysia; 5 Malaysian Research Institute on Ageing (MyAgeing), Universiti Putra Malaysia, Serdang, Selangor, Malaysia; 6 Faculty of Medicine and Health Sciences, UCSI University, Kuala Lumpur, Malaysia; Wroclaw University of Environmental and Life Sciences, POLAND

## Abstract

Motor vehicles emit most Malaysian PAHs in particulate matter of 2.5 μm (PM_2.5_-bound PAHs). Although traffic-related air pollution harms healthy people, there is a knowledge gap regarding PAHs’ effects on Malaysians. This study examines PM_2.5_-bound PAH concentrations, distribution, sources, and health risks in Malaysia’s high and low-traffic zones. Kuala Lumpur (KL) and Hulu Langat (HL) exhibit Malaysia’s high- and low-traffic areas. The high-volume air sampler collected 40 ambient PM_2.5_ samples at both locations. Solid-phase extraction and gas chromatography-mass spectrometry (GC-MS) assessed PAHs. The mean PM_2.5_-bound PAH concentrations in KL (5.85 ng m^-3^) were significantly higher than in HL (0.55 ng m^-3^) (p<0.001). KL has nine times more low-molecular-weight PAHs (LMW-PAHs) (2.63 vs. 0.27 ng m^-3^) and eleven times more high-molecular-weight PAHs (HMW-PAHs) (3.22 vs. 0.28 ng m^-3^) than HL. Over 51% of PM_2.5_ air samples at both sites included HMW-PAHs. Source apportionment tools (Diagnostic ratio, positive matrix factorization, and principal component analysis) showed that fossil fuel combustions (petrol and diesel) produced the greatest PAHs in both locations. Moreover, PAH exposure impinged higher carcinogenic health risks in KL than in HL. In conclusion, traffic and automobile pollution account for the short- and long-term health risks posed by PAHs in both regions.

## 1 Introduction

Polycyclic aromatic hydrocarbons (PAHs) are organic mixtures of chemically related, mainly colorless congeners [1, 2]. PAH compounds represent a common group of many chemicals and are present in the environment in different structures and toxicity [3]. PAHs are primarily emitted into the ambient air as a result of the partial burning of organic substances. There are two combustion sources: the natural sources involve volcanic eruptions and forest fires, and the anthropogenic sources include vehicle emissions, power plants, agricultural fires, and industrial production [4]. Also, most of these sources are established in or close to urban areas; therefore, PAHs are present in higher concentrations in the urban atmosphere than in the rural atmosphere [5].

The release of complex compounds of PAHs into the environment can cause serious health issues for the population. The human body is prone to PAHs through breathing, intake of food, and skin contact in general and occupational environments. Sometimes, multiple routes simultaneously affect the human body, such as inhalation and cutaneous exposure to contaminated air [6]. Several urban areas may face public health issues from atmospheric pollution due to disease sequels. Ambient air pollution causes about 4.2 million deaths worldwide, according to the World Health Organization (WHO) [7]. In the United States (US), several millions of premature deaths are attributable to traffic-related particulate matter of ≤2.5μm diameter (PM_2.5_) [8]. Meanwhile, in Malaysia, deaths attributed to PM_2.5_ increased by nearly 30% in the last 10 years [9]. In 2019, as many as 10,600 people in Malaysia were estimated to have died as a result of air pollution [10].

Based on the Malaysian Environmental Quality Report in 2016, the primary source of PAHs in metropolitan areas of Malaysia is emissions from motor vehicles. In urban regions, road transport is responsible for more than 70% of air pollution, making it the second greatest contributor to Malaysia’s total carbon dioxide emissions in 2016, accounting for 21% of the overall emissions [11]. Furthermore, motor vehicles cause 64.72% of Malaysia’s ambient pollution [12]. According to the Road Transport Department (RTD), Malaysia recorded 31.6 million units at the end of 2020, with a 10.1-million-unit growth over the past decade [12]. As Malaysia’s 2020 population was about 33.2 million, this indicates about one motor vehicle per person. In particular, Kuala Lumpur (KL), which is the capital of Malaysia, shows that vehicular emission is the predominant source of atmospheric PAHs based on the distribution pattern of these PAHs [13].

The main goal of this study was to collect ambient PM_2.5_-bound PAHs samples from Malaysian locations with different traffic levels. It is intended to determine if traffic volume affects the levels and distribution patterns of PM_2.5_-bound PAHs in urban and rural areas. The associations between the PAH compounds themselves, as well as their associations with meteorological conditions, were explored. The sources behind the PAHs emissions were analyzed using various approaches such as diagnostic ratio (DR), positive matrix factorization (PMF), and absolute principal component score-multiple linear regression (APCS-MLR). Additionally, BaP equivalent (BaP_eq_), lifetime lung cancer risk (LLCR), and incremental lifetime cancer risk (ILCR) indices were calculated and compared to determine the health risks of the PAHs discovered in the atmosphere. Thus, this work elucidates the concentrations, distribution, origins, and health risks of PM_2.5_-bound PAHs in Malaysia’s high and low-traffic volume areas.

## 2 Materials and method

### 2.1 Description of study areas

According to the annual report of road traffic volume in Malaysia (RTVM, 2019), Klang Valley has both high traffic volume (Kuala Lumpur) and low traffic volume (Hulu Langat) areas. Klang Valley is an urban agglomeration centered on the federal capital of Malaysia, which is Kuala Lumpur, and encompasses the state of Selangor’s neighboring cities and towns. The first study area was located at Rakyat Condominium in Bukit Kerinchi, Kuala Lumpur. Kuala Lumpur serves as the country’s primary financial, commercial, and industrial hub. It also has a very high traffic density. This sampling location is about 1.15 km from the Petaling Jaya Station No. BR807 (N 3.11197 and E 101.656125), which reported the greatest daily average traffic volume of 260,288 vehicles within a 16-hour period in the last seven years, as illustrated in S1 Table. This metropolitan region is around 6 km away from the heart of Kuala Lumpur, and the region is primarily residential and industrial, with dense road traffic. The location of this study is highly close to the busiest highways in the country, including the North-South Expressway, the Kuala Lumpur-Seremban Expressway, and the New Pantai Expressway. Twenty PM_2.5_ specimens were gathered at the entrance of Rakyat Condominium, Kuala Lumpur, in March 2021.

**Fig 1:**
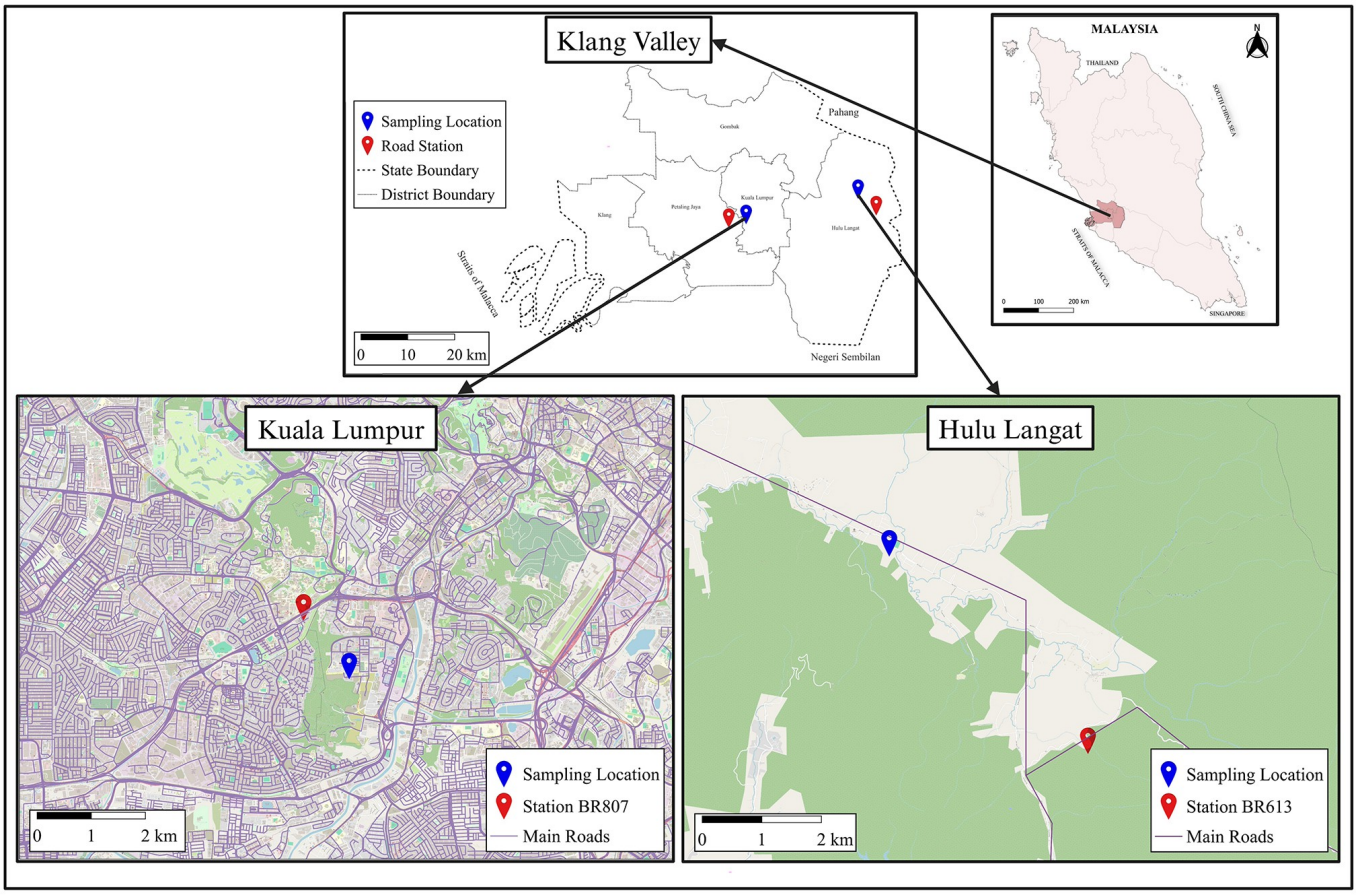
Map of study locations [The map showed both study areas in Klang Valley (dark pink area), Malaysia. The dark blue symbols referred to the sampling locations].

The second study area was located near the community center of Sungai Lui Village in Hulu Langat, Selangor. This village is a rural area with low-density road traffic, as it is far from the main highways and about 20.3 km from the heart of Kuala Lumpur. The sampling station of PM_2.5_ samples was at the ground very close to Sungai Lui Street and is about 5 km from the Hulu Langat Station No. BR613 (N 3.13398 and E 101.91613), which reported the lowest daily average traffic volume of 775 vehicles within a 16-hour period in the last seven years, as shown in S2 Table. Similarly, 20 air samples were gathered at Sungai Lui Village in April 2021. These two sampling areas are shown in Fig 1.

### 2.2 Air-mass back trajectories

The ARL HYSPLIT (version 5.3.2) model of the National Oceanic and Atmospheric Administration (NOAA) was employed to assess air-mass back trajectories. Meteorological data was acquired from the Global Forecasting System (GFS). For each 6-hour interval during the sampling periods, 80 frequencies of back trajectories calculated at the 500 m level were estimated [14].

### 2.3 PM_2.5_ field sampling

The average temperature in the Kuala Lumpur area was 32.2°C with a range of (26–35°C) while it was 28.6°C with a range of (25–30°C) in the Hulu Langat area [15, 16]. The minimum relative humidity was 44% in the Kuala Lumpur area and 70% in the Hulu Langat area. However, the maximum relative humidity was 89% in both areas, with an average of 61.35% and 74.95% in the Kuala Lumpur and Hulu Langat areas, respectively. Wind speed was, on average, 12 km h^-1^ in Kuala Lumpur, which is about double the wind speed of Hulu Langat 6.25 km h^-1^. The wind direction during the sampling period frequently fluctuates between the southwest, west, and northwest as a result of the first inter-monsoonal period, which typically takes place between March and April and is characterized by a greater degree of variability in wind directions than the monsoon seasons. The meteorological conditions during air sampling at Kuala Lumpur and Hulu Langat in 2021 were illustrated in the S3 and S4 Tables, respectively.

The PM_2.5_ high-volume air sampler (Ecotech HiVol 3000) was utilized to gather air samples on quartz microfiber filters (20.3 x 25.4 cm, catalog number 1851865) (Whatman, United Kingdom) at a flow rate of 0.83 m^3^ min^-1^ for 24 hours [17]. Firstly, these filters were pre-treated using an AS ONE (SMF-1, Yamato Scientific, Vietnam) electric furnace at 500 ºC for three hours to get rid of any deposited organic contaminants. Secondly, to ensure the consistency of mass concentration, the unused filters were kept at room temperature in the desiccator, which contained silica gel, over 24 hours, followed by weighing and wrapping them in aluminum foil to avoid any contamination. Similarly, the exposed filter papers were covered with aluminum foil to inhibit photodegradation and kept in the desiccator for 24 hours preceding the post-sampling weighing. A five-digit GR-202 semi-micro high-resolution electronic analytical balance (A&D, Japan) was utilized to weigh these filter papers. Lastly, these exposed filter papers were kept at 4 ºC until the date of PAHs extraction.

### 2.4 Extraction and analysis of PM_2.5_-bound PAHs

The exposed filters were sliced into small pieces and immediately deposited in 50 mL glass centrifuge tubes with one ppm of Chrysene-D12 and Phenanthrene-D10 (Sigma-Aldrich, USA) as surrogate standards, according to Khan et al. (2015) [17]. Twenty milliliters of dichloromethane (DCM) (Merck, Germany) were applied to the tube containing filter pieces. The process of extraction included three procedures: ultrasonic vibration, centrifuging, and mechanical shaking. The centrifuge tubes were sonicated in an ultrasonic bath (LeelaSonic—150, India) for 20 mins, and the temperature was maintained below 30°C by utilizing an ice bath. These tubes were then centrifuged at 2500 rpm (MPW—352R, Poland) for 10 mins before shaking using a vortex mixer for 10 mins. Before filtering the extract via glass microfiber filters manufactured by Whatman (United Kingdom), the sonication and centrifugation processes were carried out a total of three times.

The filtrate was concentrated to 200 μL using nitrogen gas prior to the addition of 800 μL of n-hexane (Merck, Germany). The PAH extracts were cleaned up and pre-concentrated using silica solid phase extraction (SPE) cartridges (Lichrolut^®^ RP-18, Merck, Germany). Ten milliliters of n-hexane were used for the conditioning of these RP-18 cartridges. Under a gentle vacuum, the extracts were loaded and passed through the RP-18 cartridges. At a flow rate of 1 mL min^-1^, the targeted compounds were eluted using a mixture of DCM and n-hexane (1:9 ratio). The eluates were gathered inside 20 mL glass centrifuge tubes, and their volumes were reduced to 500 μL via a gentle stream of nitrogen gas. The extract was then diluted to 1.5 mL using n-hexane in a glass vial for quantification by gas chromatography-mass spectrometry (GC-MS) (Agilent, 5975C, USA).

In conjunction with the GC-MS analysis, an HP-5MS capillary column (internal diameter 0.25 mm, 30 m length, and 0.25 mm thickness) was used. For data collection, the selected ion monitoring (SIM) technique was utilized instead of full scan mode to ensure extra sensitivity. External calibration alongside PAHs standard mixture (AccuStandard, USA) was applied to quantify all 16 PAHs. These 16 PAHs were naphthalene (NAP), acenaphthene (ACP), acenaphthylene (ACY), fluorene (FLR), phenanthrene (PHE), anthracene (ANT), fluoranthene (FLT), pyrene (PYR), benzo(a)anthracene (BaA), chrysene (CHR), benzo(b)fluoranthene (BbF), benzo(k)fluoranthene (BkF), benzo(a)pyrene (BaP), indeno[1,2,3-cd]pyrene (IcP), dibenzo[h]anthracene (DhA), and benzo[g,h,i]perylene (BgP).

### 2.5 Quality control

When the extraction procedure began, all PM_2.5_-bound PAHs samples were spiked with one ppm of the surrogate standards Chrysene-D12 (Sigma-Aldrich, USA) and Phenanthrene-D10 (Sigma-Aldrich, USA). The mean recoveries were 88.18% and 89.22% for Chrysene-D12 and Phenanthrene-D10 in the PM_2.5_-bound PAHs samples, respectively. The recovery ranges for Chrysene-D12 and Phenanthrene-D10 were 74.46 to 101.9% and 79.34 to 99.1%, respectively.

The concentrations of all 16 PAHs were not adjusted based on the average recoveries of surrogate standards of chrysene-D12 and phenanthrene-D10. For each PAH, we determined the limit of detection (LOD) to be 3 times the standard deviation of 8 replicates. The S5 Table displayed the predicted LOD for each PAH. Each set of samples underwent the same procedure, including the addition of a spiked field blank sample for analysis. PAH standards were used to evaluate the efficacy of the extraction and cleanup processes. One laboratory blank sample with only surrogate PAH standards was run with each sample patch to monitor contamination during extraction and analysis. The calibration curves for peak areas and concentrations of sixteen PAH standards (AccuStandard, USA) showed linear correlations with coefficients of determination (R^2^ > 0.99).

### 2.6 Source apportionment approaches

#### 2.6.1 Diagnostic ratio

DR is an essential qualitative method to identify sources behind PAHs emissions. In the context of environmental monitoring or toxicology, the DRs of PAHs refer to the proportion of specific PAH congeners present in a sample. These ratios can be used to identify potential sources of PAH contamination and provide information about the origin and composition of the PAHs mixture in the environment.

#### 2.6.2 Positive matrix factorization (PMF)

The United States Environmental Protection Agency (USEPA) used the PMF 5.0 model to assess PAH sources [18]. PMF reduces outlier effects on contribution and profile fits, making it an effective multivariate receptor approach. PMF uses error estimation, non-negative factor scores, and loadings compared to principal component analysis (PCA) [19]. After pre-treatment and organic composition validation, the PMF model was run. This model uses a mass balance Eq (1) to estimate source profiles and contributions:

$${\text{X}}_{\text{ij}}=\sum_{\text{k}=\text{1}}^{\text{p}}\;{\text{g}}_{\text{ik}}{\text{f}}_{\text{kj}}\text{+}{\text{e}}_{\text{ij}}$$ (1)

where X_ij_ represents the ith component concentration, source k’s contribution is denoted by g_ik_, its concentration is f_kj_, and the residual for each specimen is e_ij_. Finding the optimal combination of g_ik_ and f_kj_ to reproduce X_ij_ is the primary objective. Minimizing the Q value determines the values of g_ik_ and f_kj_. Q represents the goodness of fit for the PMF model and is defined by Eq (2) as the sum of squares of residuals with data point error estimates (S_ij_) weighted inversely:

$$\text{Q}=\sum_{\text{j}=\text{1}}^{\text{m}}\sum_{\text{i}=\text{1}}^{\text{n}}\;\frac{{\text{e}}_{\text{ij}}^{\text{2}}}{{\text{s}}_{\text{ij}}^{\text{2}}}$$ (2)

PMF requires two files for each sample: 1) concentration and 2) uncertainty. To ensure accuracy, noise, missing data, and levels below the method’s detection limit were filtered from each PAH concentration. Outlier variables are deleted. Half of the median value was substituted for missing data, and half of the lowest detectable level was substituted for compounds below the method detection limit (MDL). The other file contains the uncertainty values associated with every variable for every specimen. The authors used empirical Eqs (3) and (4) to assess the magnitude of error associated with the compound concentrations [17].

$$\sigma_{\text{ij}}=0.01({\text{X}}_{\text{ij}}+{\text{X}}_{\text{j}})$$ (3)

$${\text{S}}_{\text{ij}}=\sigma_{\text{ij}}+{\text{CX}}_{\text{ij}}$$ (4)

where σ_ij_ is the expected measurement error for the jth compound in the specimen, X_ij_ is the observed concentration, X_j_ is the average value, S_ij_ is the uncertainty, and C is a constant. C was set at 0.2 because it had the lowest percentage error and Q_true_/Q_exp_ (0.58). The Q_true_/Q_exp_ ratio measures how well the PMF model fits observed data relative to theoretical expectations.

To accommodate gravimetric mass test, filter paper preparation, and calibration curve creation errors, 5% uncertainty was introduced [17, 20]. This study identified 16 congeners as strong and the total PAHs content as ’weak’ to minimize any effect on procedure efficacy. The factor’s number was changed to increase true Q above theoretical Q. The factor’s number was changed to evaluate PMF response to 3, 4, 5, and 6 factors using key outcomes. First, lowest Q_true_ or Q_exp_; second, bootstrapping mapping into base factors; third, bootstrapping errors; and fourth, compound regression coefficient (r). After extensive testing, we defined three factors as PMF inputs based on the lowest Q_true_/Q_exp_ = 1.67, and more than 90% of the base factors align with the factors generated from a bootstrap analysis with 100 iterations and a minimum correlation threshold of 0.6.

#### 2.6.3 Absolute principal component score-multiple linear regression (APCS-MLR)

The collected data were analyzed statistically using SPSS statistical software program version 27 (SPSS Inc., USA). In the present study, all values above the detection threshold, and all compounds were found in 100% of ambient samples. PCA helps clarify data and discover variables or factors that explain correlations. PCA reduces data to a few factors that explain most of the variance in several manifest variables. The absolute principal component score-multiple linear regression (APCS-MLR) framework was used to assess pollutant source contributions using PCA, covariance matrix, varimax rotation, and MLR [21, 22].

To reduce concentration variability, the initial phase involved subtracting the concentration of each PAH congener from its mean value and subsequently dividing the result by its standard deviation according to Eq (5):

$${\text{Z}}_{\text{ik}}=\frac{{\text{C}}_{\text{ik}}–{\text{μ}}_{\text{i}}}{\sigma_{\text{i}}}$$ (5)

where Z_ik_ is the standardized Z-score of the concentration C_ik_, and the latter represents the concentration of compound i in sample k, μ_i_ is the arithmetic mean concentration of compound i, and σ_i_ is the standard deviation for compound i [22, 23].

The PCA methodology was implemented on the normalized dataset. Securing a suitable PC factor model constituted the second phase and the most formidable challenge. Our alternatives included modifying the PC factor count, the Eigenvalue threshold, and the PC loading rotation solution. To obtain the lowest group of PCs with the highest variability, the steps described by Jamhari et al. [13] and Chiu et al. [22] were used [13, 24]. The Kaiser-Meyer-Olkin (KMO) test determines whether PCA is acceptable. PCA can be applied to the dataset if the KMO measure approaches one. A KMO value less than 0.6 might suggest that the given data set is not appropriate for PCA [13]. Our study’s KMO value is 0.70, indicating that the dataset is appropriate for the PCA procedure.

Therefore, PCA was used to determine the origins of PAHs in our research areas. In the next phase, determine how much each predicted source contributed quantitatively. Two procedures were done for this phase. An artificially "zero sample" method assumed that all PAH congener concentrations were zero, adjusting PCA scores for each PC [22, 25]. Each PAH congener’s artificial zero is calculated by dividing its negative mean by its standard deviation. The estimation of APCS scores involved reducing the negative indices in each component score by subtracting the observed factor scores of actual samples (PCA scores) from the artificial zero sample. The total PAHs concentration was regressed against these APCS to calculate the overall estimation of the coefficient and, thereby, the contribution of each factor using the following Eq (6):

$${\text{Y}}_{\text{i}}={\text{X}}_{0\text{i}}+\sum_{\text{j}=1}^{\text{p}}{\text{X}}_{\text{ij}}\;{\text{APCS}}_{\text{i},\text{j}}$$ (6)

where Y_i_ is the measured concentration of the samples for element i, X_0i_ represents the constant of the MLR for element i, and p is the number of sources. X_ij_ denotes the coefficient of the source associated with component j for element i, and APCS_i,j_ denotes the rotated absolute component score for component j pertaining to element i. The (Xij APCS_i,j_) represents the contribution of source j to element i, as determined by the component labeled as j [26].

### 2.7 Carcinogenic health risk assessment

The WHO endorses BaP as the best indicator of airborne PAH carcinogenicity and uses it as a reference compound in relevant studies [27]. Most PAH toxicity and exposure knowledge comes from BaP, the best-studied PAH. Moreover, BaP alone may underestimate atmospheric PAH mixtures’ cancerous potential because the accompanied compounds are cancerous, too [27]. The BaP equivalent (BaP_eq_) value was computed by multiplying the mass concentration of specific PAH species by their toxic equivalent factor (TEF) [28] as in Eq (7):

$$\begin{array}{l} \text{Ba}{\text{P}}_{\text{eq}}=0.001\left(\text{NAP}+\text{ACE}+\text{ACP}+\text{FLR}+\text{PHE}+\text{FLT}+\text{PYR}\right)+\\ 0.01\left(\text{ANT}+\text{CHR}+\text{BgP}\right)+0.1\left(\text{BaA}+\text{BbF}+\text{BkF}+\text{IcP}\right)+\text{BaP}+\text{DhA} \end{array}$$ (7)

TEF for PAHs relied on a vital research [29]. The calculations of the lifetime lung cancer risk (LLCR) for all PAHs were performed using the provided Eq (8):

$$\text{LLCR}=\sum {\text{BaP}}_{\text{eq}}{\text{X}\;\text{UR}}_{\text{BaP}}$$ (8)

The inhalation cancer unit risk (UR_BaP_) for PAH compounds has been endorsed by the WHO and is reported to be 8.7 × 10^−5^. The inhalation route was also employed to assess the exposure to PAHs linked to fine particulate matter to determine the lifetime average daily dose (LADD) and the incremental lifetime cancer risk (ILCR) of PAHs according to Eqs (9) and (10):

$$\text{LADD}=\frac{\text{C}\;\text{x}\;\text{IR}\;\text{x}\;\text{ED}\;\text{x}\;\text{EF}}{\text{BW}\;\text{x}\;\text{ALT}}$$ (9)

ILCR=LADDXCSF (10)

where C = PAHs concentration in PM_2.5_ (mg m^−3^); IR = air inhalation rate (20 m^3^ day^-1^); ED = lifetime exposure duration (52 years); EF = exposure frequency (350 days year^-1^); BW = body weight (70 kg); ALT = average lifetime for carcinogens (70 years × 365 days year^-1^ = 25, 550 days); CSF = cancer slope factor (3.14 mg kg^−1^ day^−1^ for BaP from inhalation) for adult population [20, 30, 31].

### 2.8 Statistical analysis

For statistical analysis, version 27 of IBM SPSS Statistics was utilized. An analysis was conducted on the dataset to identify any missing data or outliers. The normality test examines parameter distribution using the Shapiro-Wilk, Kolmogorov-Smirnov, and Skewness statistics. Mean, standard deviation (SD), and range (minimum and maximum values) were employed to assess continuous variables that followed a normal distribution. In contrast, to assess continuous variables with an abnormal distribution, the median and interquartile range were employed. Using an independent t-test, the PM_2.5_-bound PAHs of KL and HL areas were compared with respect to their normal distribution. Furthermore, Pearson’s correlation analysis was employed to examine the relationships between ambient PAH congeners.

The 16 PAHs were classified based on their molecular weight and the number of benzene rings. NAP, ACP, ACY, FLR, PHE, ANT, FLT, and PYR were classified as low molecular weight PAHs (LMW-PAHs), whilst the remaining eight compounds (BaA, CHR, BbF, BkF, BaP, IcP, DhA, and BgP,) were classified as high molecular weight PAHs (HMW-PAHs) [32]. In addition, we categorized the 16 PAHs compounds according to the number of benzene rings in their structures into those with four and more rings (≥4 rings) and those with fewer than four rings (<4 rings). The ≥4 ring PAHs comprised of HMW-PAHs together with FLT and PYR, whereas the <4 ring PAHs encompassed the remaining six compounds.

The traffic data (types of vehicles: light motor vehicles, heavy motor vehicles, and total vehicles) was obtained from the Malaysian Ministry of Works to investigate the relationship between the types of motor vehicles and the emission of PM_2.5_-bound PAHs. The 16-hour traffic volume was chosen over the 24-hour traffic volume due to the fact that the Malaysian Ministry of Work’s data did not include the 24-hour traffic volume for certain stations, whereas the 16-hour traffic volume data was available for all stations in the country. Consequently, we employed the 16-hour traffic volume to compare the traffic volume data of all stations in Malaysia. This method allowed us to determine that Kuala Lumpur and Hulu Langat have the highest and lowest traffic volumes, respectively.

Additionally, our findings were not influenced by the fact that the 24-hour and 16-hour traffic volumes differed by less than 5% in both regions. Subsequently, we documented the traffic volume during the 16-hour period in the current study. In the Kuala Lumpur sampling site, there were an average of 214,998 light vehicles, 45,290 heavy vehicles, and 260,288 total vehicles. In contrast, the Hulu Langat sampling site had an average of 499 light vehicles, 276 heavy vehicles, and 775 total vehicles.

Although we possess only annual average traffic volume data for each sample site, our objective necessitated the division of this information into a 20-day period by presuming a uniform daily distribution at each location. This is based on the fact that these two sampling locations show the highest and lowest traffic volume consistently throughout the last seven years. Additionally, we employed the identical vehicle composition to assess (light vs. heavy vs. total vehicles) across the sampling period in both study locations. Consequently, we obtained two sets of daily average traffic volume and vehicle composition data across the sampling period of 20 days at both study sites. Next, PM_2.5_-bound PAHs were categorized into two-ring, three-ring, four-ring, five-ring, and six-ring. Using sensitivity analysis, which utilized the one-factor-at-a-time (OAT) technique and standardized regression determination coefficient (R^2^) values of multiple linear regression, we examined the relative impact of each of the five PAH chemical categories concerning the different vehicle types.

## 3. Results and discussion

### 3.1. Concentrations of atmospheric PM_2.5_

**Fig 2:**
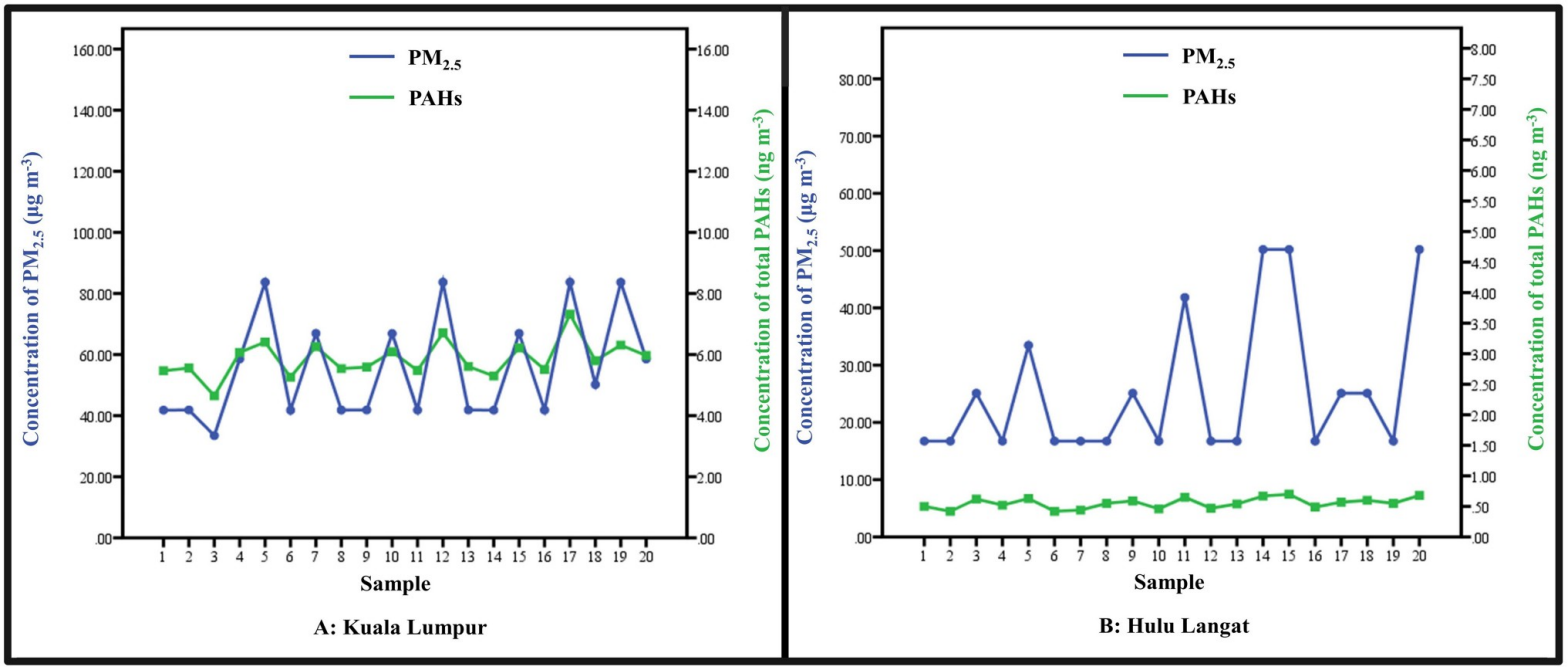
Daily sample concentration of PM_2.5_ and total PAHs in, A: Kuala Lumpur, B: Hulu Langat.

The air masses’ back trajectories at the sampling locations are depicted in S1 Fig. In general, the HYSPLIT model demonstrated that the local air masses were the primary factor influencing the sampling locations during the air sampling dates (March and April 2021). The trends of PM_2.5_ concentrations in both study locations are illustrated in Fig 2. In Kuala Lumpur, PM_2.5_ concentrations ranged from a minimum of 33.47 μg m^-3^ to a maximum of 83.71 μg m^-3^. The minimum PM_2.5_ concentration in Hulu Langat was recorded as 16.73 μg m^-3^, while the maximum concentration was 50.2 μg m^-3^. In comparison to the mean level in HL (25.51 ± 2.81 μg m^-3^), the average concentration of PM_2.5_ in KL (55.63 ± 3.9 μg m^-3^) was twice as high. The PM_2.5_ concentration in HL was below the New Malaysia Ambient Air Quality Standard (NMAAQS) limit of (35 μg m^-3^) [33], however, the KL concentration of PM_2.5_ exceeded that limit. The PM_2.5_ findings of this study were greater than those reported by Abdullah et al. (2020) during the Malaysia Movement Control Order (MCO) I and II [34]. The MCO I and MCO II in Malaysia imposed a number of restrictions on large-scale gatherings and vehicle movement, the entrance of foreign nationals and tourists, and the closure of educational institutions, governmental, and private agencies [35]. As a result of these MCOs, the PM_2.5_ concentrations significantly dropped in Malaysia [34]. On the contrary, our samples were collected directly after lifting the MCO II and returning to the usual life, which may explain the higher levels of PM_2.5_ in this study.

### 3.2. Concentrations of PM_2.5_-bound PAHs

Ambient air samples in Kuala Lumpur and Hulu Langat detected 100% of PAHs congeners in the PM_2.5_ phase. The outcomes of this study revealed that the mean of each PAH pollutant, LMW-PAHs, HMW-PAHs, and total PAHs were ultimately greater in Kuala Lumpur than in Hulu Langat areas (Table 1). The mean of total PAHs in Kuala Lumpur was 5.85 ± 0.59 ng m^-3^, which was ten times greater than in Hulu Langat at 0.55 ± 0.08 ng m^-3^. This trend shows that urban areas like Kuala Lumpur have massively elevated levels of PAHs in comparison to rural areas like Hulu Langat. These higher concentrations of PAHs in Kuala Lumpur may partly be attributed to the elevated population density and industrial activity in this urban area. In India, one of the Southeast Asian countries, the urban areas showed the highest concentrations of total PAHs, while the rural areas had the lowest concentrations throughout the sampling duration [36].

**Table 1:** Mean, SD, and independent t-test outcomes of PM_2.5_-bound PAHs in Kuala Lumpur and Hulu Langat.

PAH Compound		KL (n = 20)		HL (n = 20)		T value	Mean difference	P-value
		%^a^	Mean ± SD (ng m^-3^)	%^a^	Mean ± SD (ng m^-3^)			
**LMW-PAHs**	**NAP**	100	0.44 ± 0.08	100	0.04 ± 0.005	21.79	0.41	<0.001
	**ACP**	100	0.25 ± 0.07	100	0.01 ± 0.005	14.63	0.25	<0.001
	**ACY**	100	0.23 ± 0.05	100	0.03 ± 0.005	17.95	0.22	<0.001
	**FLR**	100	0.37 ± 0.06	100	0.03 ± 0.005	25.15	0.37	<0.001
	**PHE**	100	0.34 ± 0.07	100	0.02 ± 0.005	19.36	0.33	<0.001
	**ANT**	100	0.33 ± 0.07	100	0.06 ± 0.005	20.69	0.33	<0.001
	**FLT**	100	0.34 ± 0.09	100	0.04 ± 0.005	15.18	0.34	<0.001
	**PYR**	100	0.32 ± 0.10	100	0.04 ± 0.005	13.62	0.32	<0.001
	**LMW-PAHs**	100	2.63 ± 0.28	100	0.27 ± 0.04	37.54	2.36	<0.001
**HMW-PAHs**	**BaA**	100	0.35 ± 0.07	100	0.05 ± 0.005	20.94	0.33	<0.001
	**CHR**	100	0.66 ± 0.14	100	0.05 ± 0.005	14.16	0.63	<0.001
	**BkF**	100	0.33 ± 0.01	100	0.04 ± 0.005	4.83	0.02	<0.001
	**BaP**	100	0.45 ± 0.18	100	0.04 ± 0.005	3.51	0.02	0.001
	**BbF**	100	0.67 ± 0.18	100	0.04 ± 0.005	15.65	0.66	<0.001
	**IcP**	100	0.47 ± 0.14	100	0.04 ± 0.005	13.79	0.46	<0.001
	**DhA**	100	0.18 ± 0.08	100	0.01 ± 0.005	9.34	0.17	<0.001
	**BgP**	100	0.14 ± 0.03	100	0.01 ± 0.005	10.37	0.01	<0.001
	**HMW-PAHs**	100	3.22 ± 0.45	100	0.28 ± 0.04	28.7	2.94	<0.001
	**Total PAHs**	100	5.85 ± 0.59	100	0.55 ± 0.08	39.67	5.30	<0.001

Abbreviation:

^a^: Detection percentage KL: Kuala Lumpur HL: Hulu Langat SD: Standard Deviation

BbF was reported as the PAH congener with the highest mean level of 0.67 ± 0.18 ng m^-3^ in the PM_2.5_ air samples of Kuala Lumpur. The BbF is a vital tracer of vehicular emissions [37]. In tandem with our findings, research by Khan et al. (2015) revealed that BbF was the most abundant PAH congener (0.57 ng m^-3^) in Malaysia [17]. ANT was the PAH congener of the highest mean level of 0.06 ± 0.005 ng m^-3^ in the PM_2.5_ air samples of Hulu Langat. ANT is one of the common PAH compounds linked to traffic emissions [38]. However, BgP represented the PAH compound with the lowest mean level among the PM_2.5_ air samples of Kuala Lumpur and Hulu Langat, with values of 0.14 ± 0.03 and 0.01 ± 0.005 ng m^-3^, respectively. Some studies reported that BgP could be a central tracer of diesel exhausts [39–41].

Table 2 summarizes the results of PAHs in PM_2.5_ and PM_10_ from selected studies in Malaysia. Overall, the concentration of total PAHs at Kuala Lumpur in our study is greater than the majority of these studies. The overall levels of PM_2.5_-bound PAHs increased over time as a result of many contributors, including an increase in the size of the population, the number of vehicles, levels of urbanization and industrialization, weather conditions, and geographic regions. However, the concentration of total PAHs at the Hulu Langat site was the lowest among all these Malaysian studies. This can be explained by the fact that Hulu Langat is a rural area with low traffic volume in Peninsula Malaysia. Also, this is the first study to measure the ambient PM_2.5_-bound PAHs in this region.

**Table 2:** Ambient PAHs concentrations in Malaysia.

Location	Location type	Year of sampling	PM fractions	Conc. (ng m^-3^)	References
Kuala Lumpur	Urban	2021	PM_2.5_	5.85 ± 0.59	This study
Hulu Langat	Rural	2021	PM_2.5_	0.55 ± 0.08	This study
Kuala Lumpur	Urban	2019	PM_2.5_	1.74 ± 2.68	[42]
Kuala Lumpur	Urban	2015–2016	PM_2.5_	2.04 ± 0.28	[43]
Bangi	Semi-urban	2013–2014	PM_2.5_	2.79	[17]
Bangi	Semi-urban	2010–2011	PM_10_	2.54	[13]
Kuala Lumpur	Urban	2001	PM_10_	3.10 ± 2.92	[44]
Kuala Lumpur	Urban	1998–2000	PM_10_	6.28 ± 4.35	[45]

Table 3 illustrates the ambient PAHs concentrations reported worldwide by researchers. PAH levels measured in Australia [46], Bangladesh [47], China [48–52], Croatia [53], Iraq [54], Iran [55], Pakistan [56], and Turkey [57] were significantly greater than the levels of PAHs in the current study. Notably, the values in Japan (0.3–1 ng m^-3^) [58] are considerably lower than the values in our study (5.85 ± 0.59 ng m^-3^). The fact that only 9 out of 16 PAH compounds were measured in the Japanese study may have contributed to this difference [58]. Total ambient PAH concentrations measured in Mexico by Amador-Munoz et al. (2020) are at a comparable level (4.48 ng m^-3^) with our results [59]. However, the Mexican level of atmospheric PAHs was 5.69 ng m^-3^ about one decade ago [60], which is almost close to the current level of PAHs in Kuala Lumpur (5.85 ng m^-3^). The decrease in the concentration of total PAHs in Mexico is likely attributable to the air quality management programs implemented over the past two decades. Governmental strategies in Mexico have been proven effective in improving air quality by promoting the use of vehicles with improved combustion technology, enhanced catalytic converters, and high-quality fuels [59]. Additionally, it is well known that PAH emissions increase with engine wear, mileage, temperature (especially at freezing starts), speed, and quality of fuel [61]. It has been demonstrated that the use of catalytic converters reduces the PAH levels in vehicle emissions by a factor of 25 [62].

**Table 3:** Ambient PM_2.5_-bound PAHs concentrations worldwide.

Country	Year of air sampling	Concentrations (ng m^-3^)	References
Brisbane, Australia	2010–2012	38	[46]
Bangladesh	2017–2018	22.4±6.9	[47]
Beijing, China	2018	78±54	[48]
Harbin, China	2017–2018	86.9	[50]
Huanggang, China	2018–2019	7.35±6.79	[52]
South China Sea, China	2018	41.3±24.7	[49]
Taiyuan, China	2019	12	[49]
Zagreb, Croatia	2014	9.79±7.82	[53]
Karaj City, Iran	2018–1019	16.16–22.55	[55]
Baghdad, Iraq	2012–2013	18±12	[54]
Kanazawa, Japan	2017–2018	0.3–1	[58]
Mexico City, Mexico	2016–2017	4.48	[59]
Islamabad, Pakistan	2017	25.69±11.96	[56]
Bursa, Turkey	2021–2022	24.5±19.93	[57]

### 3.3. Distribution of PAHs according to their ring number and molecular weight

The contribution percentages of PAHs to the overall PAHs based on their number of rings were in the same descending order (4-rings > 3-rings > 5-rings > 6-rings > 2-rings) in both study areas, as illustrated in S2 Fig. In particular, the findings of both study areas revealed that the ≥4 rings PAHs formed 66% of the total PAHs, whereas <4 rings PAHs constituted 34% of the total PAHs. In addition, there were statistically significant differences (p <0.001) between each ring category of PAHs and its counterpart in both study regions. In the current study, it is shown that HMW-PAHs continue to exhibit a higher prevalence in the Klang Valley region compared to LMW-PAHs, as shown in S3 Fig. This observation aligns with prior research conducted in the same geographical region several years ago [17]. Emissions from automobiles serve as the primary contributor to air pollution in that area.

The overall mean of LMW-PAHs and HMW-PAHs in Kuala Lumpur were 2.63 ± 0.28 and 3.22 ± 0.45 ng m^-3^, respectively. However, the overall mean LMW-PAHs and HMW-PAHs in Hulu Langat were 0.27 ± 0.04 and 0.28 ± 0.04 ng m^-3^, respectively. The LMW-PAHs were nine times higher, and HMW-PAHs were eleven times higher in Kuala Lumpur than Hulu Langat. The HMW-PAHs constituted the major part (55%) of the total PAHs in the PM_2.5_ air samples of Kuala Lumpur, while the LMW-PAHs constituted the rest (45%), as shown in S3a Fig. In contrast, there was a slight difference in the contribution percentage of the total PAHs in Hulu Langat PM_2.5_ air samples between the HMW-PAHs and LMW-PAHs (51%) and (49%), respectively, as shown in S3b Fig.

The lower concentrations of LMW-PAHs in comparison to HMW-PAHs may be explained by the fact that the vapor phase is the predominant form of LMW-PAHs. Also, non-volatile HMW-PAHs are found in high concentrations in the particle phase [63]. This is a result of the comparatively low vapor pressure, which is more than sufficient to keep the high molecular weight PAHs trapped in particles as opposed to the low molecular weight PAHs [64]. Many studies have stated that HMW-PAHs were significantly greater than LMW-PAHs due to traffic-related sources, and the outcomes indicated that the emissions from vehicle exhaust, encompassing both diesel and petrol vehicles, were the main source behind the HMW-PAHs [63, 65–67].

### 3.4. Pearson’s correlations, meteorological factors, and forms of traffic dependence

The outcomes of Pearson’s correlation analysis showed that the LMW-PAH congeners correlate strongly among themselves, and the HMW-PAH congeners correlate strongly among themselves as well (S6 and S7 Tables). In addition, there was an apparent distinction between LMW-PAHs and HMW-PAHs within the individual PAHs. Positive r values indicated that the correlation between these two categories was weak. Each pair of LMW-PAHs and HMW-PAHs exhibits significantly strong correlations in both Kuala Lumpur and Hulu Langat regions (p <0.01), indicating that the PAHs of these two groups may have the same emission source.

There was a strong association between PM_2.5_ and ambient PAHs (Pearson’s r = 0.67, p <0.01). Furthermore, in both sampling areas, there was a significant inverse correlation between relative humidity and temperature (p <0.01) (S8 and S9 Tables). All PM_2.5_-bound PAH compounds were negatively correlated with temperature in Kuala Lumpur and Hulu Langat areas. This finding could be explained by the fact that any increase in temperature can lead to an increase in the photolysis rate and, thereby, a reduction in the PM_2.5_-bound PAHs. Also, relative humidity and wind speed were positively correlated with most PM_2.5_-bound PAH compounds. As the humidity percentage rises, more PAHs are adsorbed by the particles in a wet environment, indicating a direct correlation between humidity and the concentration of PAHs in PM_2.5_. Despite this, most of the variables were not significantly correlated in Kuala Lumpur and Hulu Langat.

**Fig 3:**
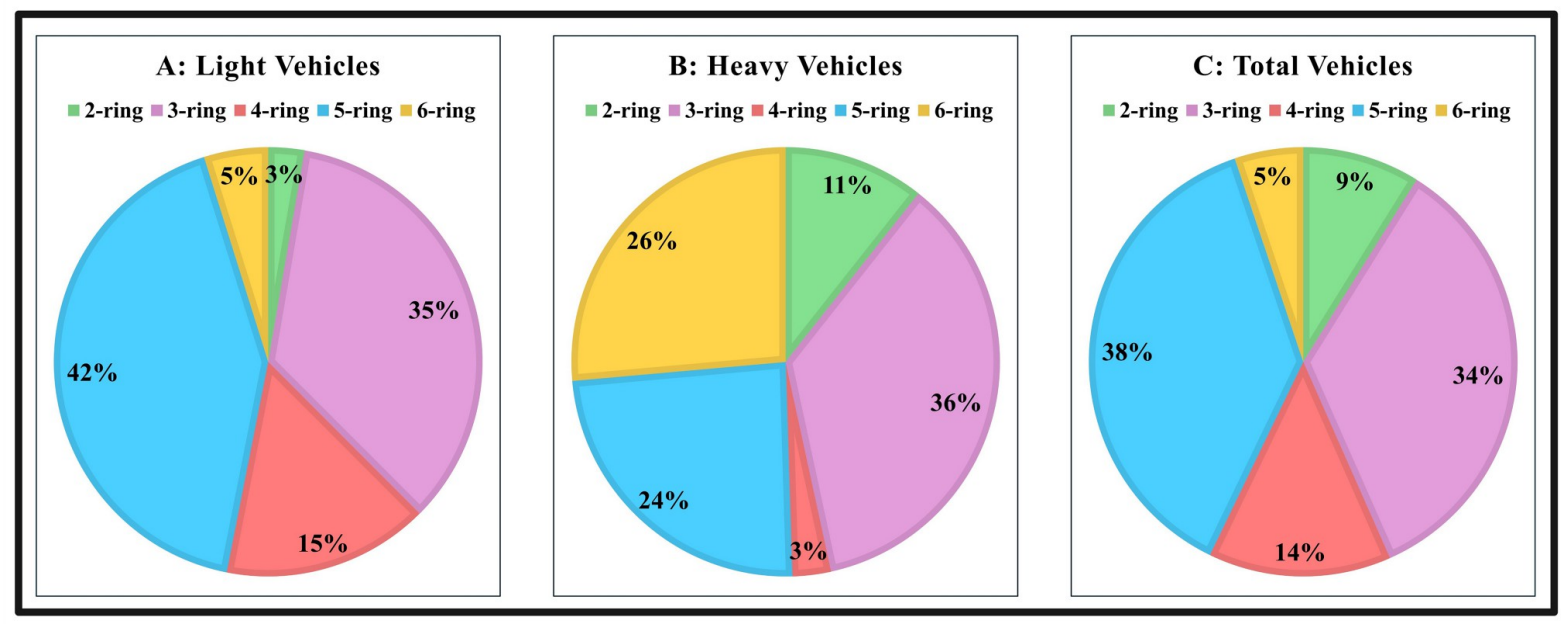
Sensitivity of PAHs to the traffic frequency by, A: Light vehicles, B: Heavy vehicles, and C: Total vehicles.

The concentrations of all except one PAH group were maintained at a consistent level, and the variation in output for each kind of vehicle (e.g., light, heavy, total vehicles) was quantified. Next, we conducted this procedure for all PAH categories to evaluate the influence of each PAH category on vehicle types. Lastly, R^2^ values are used to evaluate the overall importance of each PAH group in the model using multiple linear regression [17]. The results showed that different PAHs categories (from 2–6 rings) responded differently depending on the types of vehicles used (Fig 3). Five-ring PAHs were affected mainly by light and total vehicles (light and heavy vehicles), while six-ring PAHs were mainly affected by heavy vehicles in both study areas. LMW-, HMW-, and total PAHs were also shown to have a significant correlation with the number of light, heavy, and total vehicles (r^2^ = 0.98, p <0.001), as shown in the S10 Table.

### 3.5. Source apportionment of PM_2.5_-bound PAHs

#### 3.5.1. Diagnostic ratios (DRs)

The diagnostic ratio is an important qualitative method to identify the sources behind the PAH emissions. The DRs of the selected PAHs of both study areas are illustrated in Table 4. The DR of FLT/PYR can differentiate between sources that are petrogenic and pyrogenic. If the DR value is <1, it indicates petrogenic sources, while a value of >1 indicates pyrogenic origins [68]. The value of DR FLT/PYR was >1 in both sampling areas, providing insight into pyrogenic origins. The DR values of ANT/ANT+PHE were 0.49 and 0.76 in Kuala Lumpur and Hulu Langat, respectively. This DR indicates that a pyrogenic source predominates [69, 70]. Similarly, the primary sources of PAHS were pyrogenic sources based on the DR values of BaA/BaA+ CHR [70], which were 0.37 and 0.45 in Kuala Lumpur and Hulu Langat, respectively. Furthermore, the value of DR FLT/FLT+PYR was 0.5 in both sampling areas, and this DR points towards a fossil fuel combustion source [70].

**Table 4:** Diagnostic ratios of PM_2.5_-bound PAHs in Kuala Lumpur and Hulu Langat.

DRs	Value	Source	KL	HL	Reference
FLT/PYR	<1 >1	Petrogenic Pyrogenic	1.1	1.2	[68]
ANT/ANT+PHE	<0.1 >0.1	Petrogenic Pyrogenic	0.49	0.76	[69, 70]
FLT/FLT+ PYR	<0.4 >0.4 0.4–0.5 >0.5 0.6–0.7	Petrogenic Pyrogenic Fossil fuel Grass, wood, coal Diesel	0.50	0.50	[70]
BaA/BaA+ CHR	<0.2 0.2–0.35 >0.35 >0.5	Petrogenic Coal Pyrogenic Wood burning	0.35	0.45	[70]
IcP/IcP+BgP	<0.2 >0.2 0.2–0.5 >0.5 0.82 0.35–0.70	Petrogenic Pyrogenic Petroleum/gasoline Grass, wood, coal Oil combustion Diesel	0.70	0.70	[70]
BaP/BgP	<0.6 >0.6	Non-traffic Traffic	3.27	3.41	[70]
BaA/CHR	0.54–0.66 0.66–0.92	Industry Wood	0.56	0.83	[71]

Abbreviation: KL: Kuala Lumpur; HL: Hulu Langat; DRs: Diagnostic ratios.

The DRs of IcP/IcP+BgP and BaP/BgP are depending on larger molecules of PAHs [70]. The DR value of IcP/IcP+BgP was 0.70 in both sampling areas, indicating a diesel source. Also, the DR values of BaP/BgP were 3.27 and 3.41 in Kuala Lumpur and Hulu Langat, respectively. This could mean that the origin of HMW-PAHs is related to traffic emissions. The DR value of BaA/CHR in Kuala Lumpur was 0.56, highlighting the significant impact of industrial emissions. However, this DR value was 0.83 in Hulu Langat, which indicated a wood combustion source [71]. According to bivariate plots (S4 and S5 Figs), pyrogenic sources were the origin (100%) of the emitted PAHs in both of the study areas. In particular, fossil fuel combustion contributed 65%, and coal combustion of industrial sources contributed 35% of the PAHs levels in Kuala Lumpur. However, the combustion of fossil fuels represents the primary cause behind the PAHs levels in Hulu Langat.

#### 3.5.2. PMF

**Fig 4:**
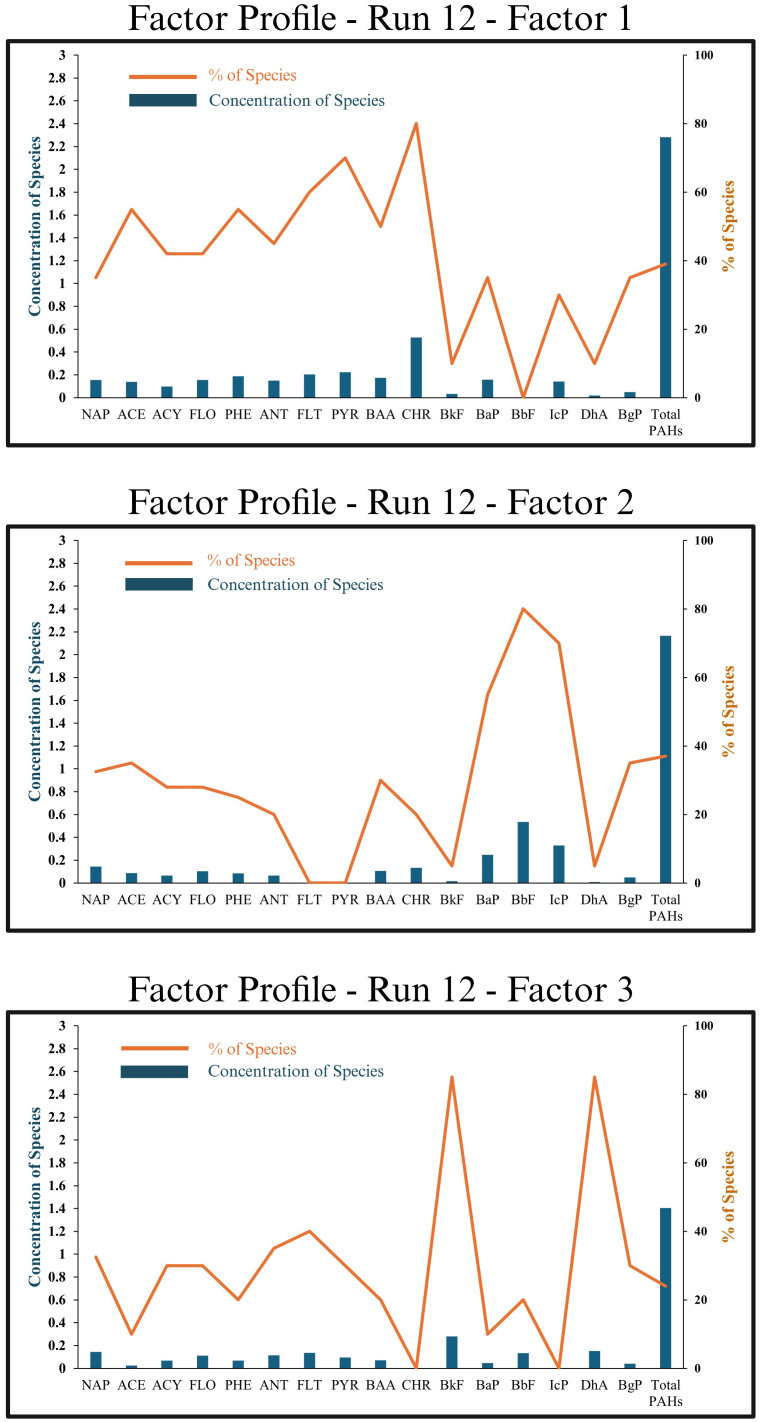
Source factor profile of PAHs identified from PMF model of Kuala Lumpur samples.

The possible sources of PM_2.5_-bound PAHs in both study areas were analyzed based on the diagnostic ratios of PAHs, as mentioned previously, which could provide a qualitative understanding of the origins of PAHs (Fig 4). However, these diagnostic markers may vary from the origin to the receptor [72], which may add ambiguity to the origin determination based solely on these diagnostic markers. Thus, the PMF model was employed to further allocate the sort and contribution of PAH sources, resulting in a three-factor solution that revealed the compositions of sources in Kuala Lumpur (Fig 4). However, the PMF model was not possible in Hulu Langat. This might be due to the smaller number of samples at this site. Despite that, these 20 samples of Hulu Langat could show one source that is compatible with the diagnostic ratios of Hulu Langat PM_2.5_-bound PAHs, which were pointing to only one factor.

According to Fig 4, factor-1 indicated coal combustion sources due to the obvious inputs of PYR (70% of PYR mass) and FLT (60% of FLT mass). This factor contributed to 39% of the PM_2.5_-bound PAHs. This could be explained as Kuala Lumpur is one of the important industrial areas in Klang Valley, and the diagnostic ratio of BaA/BaA+CHY was 0.35, indicating coal-burning sources. Coal burning was shown to be the primary contributor to FLT and PYR in a PAHs investigation done in an industrial environment [73]. Also, the molecular markers FLT and ANT were selected to determine the emission variables associated with coal burning at work [74]. Moreover, previous studies utilized a significant value of PYR and FLT as indications of coal combustion [75–77].

On the other hand, factor-2 indicated vehicular and gasoline emissions due to the heavily loaded (BaP, BbF, and IcP). This factor accounted for the largest share (around 37%) of all PAHs found in PM_2.5_. Soot from gasoline-powered vehicles has been discovered to include elevated levels of BbF, BaP, and IcP [78, 79]. Some studies found evidence that vehicle exhausts contributed to the production of BaP and IcP [63, 77].

Factor-3 indicated diesel emissions due to the heavily loaded BkF and DhA. The lowest percentage of PM_2.5_-bound PAHs came from this source, at about 24%. Soot from diesel vehicles has been shown to include an abundance of HMW-PAHs like BkF and DhA [79]. Since the site of air sampling was in the heart of an urban city where there is a constant presence of light and heavy vehicles like cars, motorcycles, vans, and lorries, even on weekends, it was expected that traffic emissions (both petrol and diesel) would account for a large percentage of PM_2.5_-bound PAHs.

Conventional PMF analysis inadequately addresses key uncertainties related to modeling flaws. Modeling error may occur if a simplified model, such as the frequently employed bivariate model, fails to accurately represent the genuine physical-chemical reality [80]. For instance, sources and processes influencing ambient PM that fluctuate over time are unlikely to be revealed by doing PMF on the complete dataset. Getting an excessive amount of trust in one source results in placing an insufficient amount of trust in others, and vice versa.

The application of fitting constraints can be employed to reduce the uncertainty associated with the rotation [80, 81]. Methods have been developed to examine the uncertainty induced by rotational ambiguity and measurement error in the source profiles [82]. Moreover, many researchers endeavored to estimate the uncertainty of source contributions; however, their methodologies were more akin to sensitivity studies than to the rigorous establishment of error boundaries [83–85].

A different approach to obtaining a dependable PMF solution is to exclude such short-term data from the initial data. However, the implementation of such exclusions would result in the loss of specific data. Our data represents the ambient concentration of both PM_2.5_ and PAHs in urban and rural areas at a specific period, which was between the MCO II and MCO III, and accurately reflects the ambient trends at that important period when the lockdown was lifted and the daily life activities resumed.

#### 3.5.3. APCS-MLR

Principal component analysis was carried out, and factor scores were calculated for the Kuala Lumpur area, as illustrated in the S11 Table. Three factors could explain 62.65% of the total data variance of Kuala Lumpur samples. However, the PCA for the Hulu Langat area was not able to identify more than one factor in a similar case to the PMF. According to the S11 Table, factor-1 (22.02% of the total data variance) was laden with NAP, FLT, PYR, BaA, and CHR. These compounds are markers of the emission of natural gas and coal combustion [13, 73, 77]. Thus, pyrogenic sources associated with coal combustion and natural gas emissions were assumed to be the primary origins of factor-1. This was in tandem with the diagnostic value of (BaA/BaA+CHR), which was 0.35, referring to the coal combustion source [24].

Factor-2 (20.85% of the total data variance) was mainly laden with PHE, ANT, BbF, IcP, DhA, and BgP. Incomplete combustion and pyrolysis of fuel are the primary sources of these HMW-PAHs (comprising 5–6 rings), which were found to be the most abundant in this factor [65]. IcP, DhA, and BgP are identified as indicators of automobile and petrol emission sources in the available literature [73]. Therefore, factor-2 was categorized as gasoline fuel combustion. Moreover, factor-3 (19.78% of the total data variance) was typically driven by BkF and BaP’s large loads. BkF was previously identified as a diesel vehicle tracer in the factor profile [64, 86]. Similarly, BaP has been extensively utilized in the field of literature as a distinctive indicator for motor vehicular emissions. Therefore, factor-3 was classified as diesel fuel combustion.

The results of the MLR analysis of the APCS contributions to the overall PAHs concentrations are illustrated in S12 Table, and their percentage contributions are shown in S6 Fig. Vehicle emissions from both petrol and diesel engines contributed equally (61.33%), whereas those from coal combustion and natural gas vehicles contributed significantly less (31.5%) to the total emissions. However, 7.17% were listed as "unaccounted for." Emissions from vehicles using petrol and diesel (3.68 ng m^-3^) are twice those of using natural gas and coal (1.89 ng m^-3^). The statistical strength of the correlation between the observed values and the MLR predictions was determined to be high (R^2^ = 0.99, p <0.01), as shown in S7 Fig.

### 3.6 Health risk assessment

Table 5 presents the BaP_eq_ data acquired in the current study, indicating a cumulative BaP_eq_ concentration in Kuala Lumpur (773 pg m^-3^) was roughly 12 times higher than in Hulu Langat areas (64.81 pg m^-3^). In both regions, the BaP_eq_ of individual BaP was >50% higher than that of overall PAHs. BaP’s BaP_eq_ was 11 times greater in Kuala Lumpur (450 pg m^-3^) than in Hulu Langat (40 pg m^-3^). This emphasizes the significance of BaP as an important compound in the evaluation of health risks associated with PAHs, providing a precise calculation of the cancerous effects [13, 43].

**Table 5:** Health risks of PM_2.5_-bound PAHs in Kuala Lumpur and Hulu Langat areas.

PAH	KL				HL			
	BaP_eq_ (pg m^-3^)	LLCR	LADD	ILCR	BaP_eq_ (pg m^-3^)	LLCR	LADD	ILCR
NAP	0.4	3.8 x10^-8^	4.4 x10^-7^	-	0.04	3.5 x10^-9^	4.0 x10^-8^	-
ACE	0.3	2.2 x10^-8^	2.5 x10^-7^	-	0.01	8.7 x10^-10^	1.0 x10^-8^	-
ACY	0.2	2.0 x10^-8^	2.3 x10^-7^	-	0.03	2.6 x10^-9^	3.0 x10^-8^	-
FLR	0.4	3.2 x10^-8^	3.7 x10^-7^	-	0.03	2.6 x10^-9^	3.0 x10^-8^	-
PHE	0.3	3.0 x10^-8^	3.4 x10^-7^	-	0.02	1.7 x10^-9^	2.0 x10^-8^	-
ANT	3.3	2.9 x10^-7^	3.3 x10^-7^	-	0.6	5.2 x10^-8^	6.0 x10^-8^	-
FLT	0.3	3.0 x10^-8^	3.4 x10^-7^	-	0.04	3.5 x10^-9^	4.0 x10^-8^	-
PYR	0.3	2.8 x10^-8^	3.2 x10^-7^	-	0.04	3.5 x10^-9^	4.0 x10^-8^	-
BaA	35	3.0 x10^-6^	3.5 x10^-7^	-	5	4.4 x10^-7^	5.0 x10^-8^	-
CHR	6.3	5.5 x10^-7^	6.3 x10^-7^	-	0.5	4.4 x10^-8^	5.0 x10^-8^	-
BkF	33	2.9 x10^-6^	3.3 x10^-7^	-	4	3.5 x10^-7^	4.0 x10^-8^	-
BaP	450	3.9 x10^-5^	4.5 x10^-7^	1.4 x10^-6^	40	3.5 x10^-6^	4.0 x10^-8^	1.3 x10^-7^
BbF	67	5.8 x10^-6^	6.7 x10^-7^	-	4	3.5 x10^-7^	4.0 x10^-8^	-
IcP	4.7	4.1 x10^-7^	4.7 x10^-7^	-	0.4	3.5 x10^-8^	4.0 x10^-8^	-
DhA	170	1.5 x10^-5^	1.7 x10^-7^	-	10	8.7 x10^-7^	1.0 x10^-8^	-
BgP	1.4	1.2 x10^-7^	1.4 x10^-7^		0.1	8.7 x10^-9^	1.0 x10^-8^	
Total PAHs	773	6.7 x10^-5^	5.8 x10^-6^		64.81	0.56 x10^-5^	0.55 x10^-6^	

In this study, the total BaP_eq_ concentration in Kuala Lumpur (773 pg m^-3^) was greater than the results of other studies conducted in the same region, such as those conducted by Jamhari et al. (2014) and Sulong et al. (2019) [13, 43]. The total BaP_eq_ concentrations reported by these two studies were 640.01 and 266.27 pg m^-3^, respectively. The concentrations of total PAHs in PM_2.5_ increased over time due to many factors, such as the continuous increase in population density, vehicle population, urbanization, and industrialization levels [17, 87, 88]. These factors are significant contributors to air pollution, and more specifically, the emission of PAHs.

This finding underscores the significance of BaP as a representative compound in the assessment of health risks posed by PAHs. Other studies have shown that BaP’s carcinogenic potency is estimated to range between 27% and 67% of the overall cancerous potency exhibited by total PAHs [17, 43]. Therefore, BaP is subjected to international regulation and has a fixed value. The WHO recommends a unit risk of BaP of 8.7x10^-5^ (ng m^-3^ per year) [7, 17]. The carcinogenicity risk of DhA, the second most potent BaP_eq_ PAH, was 22% higher in Kuala Lumpur and 15.4% higher in Hulu Langat than that of all other PAHs. The BaP_eq_ of DhA in the Kuala Lumpur samples was also 11 times higher than in the Hulu Langat samples.

The LLCR was calculated using the UR_BaP_ and the BaP_eq_ of each PAH. LLCR yielded Kuala Lumpur and Hulu Langat readings of 6.7 x10^-5^ and 0.56 x10^-5^, respectively. At the highest risk category, the annual LLCR readings must be no more than (10^−6^–10^−4^) [89]. The present study has determined that the risk of carcinogenicity associated with overall PAHs is within a tolerable range in these Malaysian regions. However, it has been observed that adult residents of Kuala Lumpur exhibit a significantly higher susceptibility to lung cancer compared to adult residents of Hulu Langat, with a 12-fold increase in risk. Accordingly, it was shown that 5–6 ring PAHs accounted for over 95% of the overall risk at both locations, which aligns with findings reported in previous studies conducted in Pakistan (90%) [30] and Japan (95%) [90]. The outcomes suggest that the presence of HMW-PAHs, specifically BaP and DhA, significantly contributed to potential health hazards. Hence, it is of ultimate significance to carry out periodic sampling of atmospheric PAHs within these regions.

The ILCR was calculated using the LADD and CSF values of BaP in the adult population, specifically, individuals aged 18 to 70 years. While CSF had a fixed value of 3.14 mg kg^−1^ day^−1^ for BaP from inhalation, the ILCR was ten times greater in Kuala Lumpur than in the Hulu Langat group. Moreover, the ILCR of the adult population was found to be greater in Kuala Lumpur (1.4 x 10^−6^) compared to Hulu Langat (1.3 x 10^−7^). This indicates that within a population of 100,000 individuals living in the Kuala Lumpur area, it is expected that 1–2 people will get cancer throughout their lifetime as a result of exposure to PAHs by inhalation. Nevertheless, an estimation has been made that the ILCR of the Hulu Langat group was at 1.3 x 10^−7^. This value suggests that within a population of one million individuals exposed to PAHs, there is a probability of 1–2 individuals developing cancer throughout their lifetime. The data presented in Table 5 indicates an increased health risk for the Kuala Lumpur group in contrast to the Hulu Lanagt group due to their comparatively greater exposure to carcinogens during an equivalent duration. The level of cancerous materials stored in the human body increases proportionally with longer exposure times. Two studies were undertaken in Kuala Lumpur by Jamhari et al. (2014) and Sulong et al. (2019) to estimate the ILCR [13, 43]. The study by Jamhari et al. (2014) reported an ILCR of 3.01×10^−7^, while the study by Sulong et al. (2019) reported an ILCR of 1.42×10^−7^ [13, 43]. The obtained data exhibited a significant disparity in comparison to our observations. This phenomenon can be attributed to the sustained growth in population, the proliferation of motor vehicles, and industrial manufacturing.

### 3.7 Strengths and limitations of the study

This study presents the first comparison of the PM_2.5_-bound PAHs levels between urban areas with the daily highest traffic volume and rural areas with the daily lowest traffic volume in Malaysia. Furthermore, this study shows the ambient concentration of both PM_2.5_ and PAHs directly after the lifting of the MCO II and the return of the usual life, which may provide more credible data that accurately reflect the ambient trends at that specific period. Additionally, for the first time, the health risk assessment compares the health risk of the Kuala Lumpur group (urban) to the Hulu Lanagt group (rural) using BaP_eq_, LLCR, and ILCR. Nevertheless, this study does possess limitations. At first, it was apparent that the total number of air samples was 40, with 20 samples from each area. In March and April 2021, we were able to obtain 40 samples. Nonetheless, the air sampling was halted in May 2021 as a result of the MCO III. Still, these 40 samples were enough to find out how traffic volume affects the concentration, pattern of distribution, associations, source apportionment, and health risk assessment of PM_2.5_-bound PAHs in both urban and rural locations.

## 4 Conclusion

The mean of total PM_2.5_-bound PAHs concentration was substantially ten times higher in the Kuala Lumpur regions (5.85 ng m^-3^) than in the Hulu Langat regions (0.55 ng m^-3^). High traffic volume (daily 260,288 vehicles per 16-hour period) and the resulting high vehicle emissions are the primary explanations for the Kuala Lumpur results, while low traffic volume (daily 775 vehicles per 16-hour period) and the resulting low vehicle emissions are the primary explanations for the Hulu Langat results. In both study regions, PAHs of ≥ 4 rings (66%) and HMW-PAHs (≥51%) were more prevalent than PAHs of <4 rings (34%) and LMW-PAHs (≤49%), respectively. The PAHs were subjected to source apportionment analysis using the diagnostic ratios, PMF 5.0 model, and APCS-MLR. This analysis successfully identified three primary pyrogenic sources, namely, the combustion of petrol and diesel, as well as the burning of natural gas and coal. The present study shows the direct relationship between atmospheric PAHs and the risk of carcinogenicity among the adult population, and vehicular emissions are the main supply of these atmospheric PAHs. The estimated BaP_eq_, LLCR, and ILCR associated with PAHs exposure in adults are tolerable in Kuala Lumpur and Hulu Langat. Nonetheless, Kuala Lumpur residents face a more significant threat to their health than those in Hulu Langat. These PAHs, specifically BaP and DhA, significantly contributed to potential health hazards and have a central role in the short and long-term adverse health effects. Therefore, it is imperative to diminish the ambient concentrations of PAHs and to conduct studies in these regions to assess the biological levels of PAHs, thereby supporting our results and monitoring the health of adult populations.

## Supporting information

S1 Table Highest traffic volume areas in Peninsular Malaysia. (DOCX)

S2 Table Lowest traffic volume areas in Peninsular Malaysia. (DOCX)

S3 Table Meteorological conditions during air sampling at Kuala Lumpur in 2021. (DOCX)

S4 Table Meteorological conditions during air sampling at Hulu Langat in 2021. (DOCX)

S5 Table LOD and matrix-standard spiking recovery of 16 PAHs. (DOCX)

S6 Table Pearson correlation coefficients (r) among the PAHs compounds analysed in PM_2.5_-bound samples of Kuala Lumpur. (DOCX)

S7 Table Pearson correlation coefficients (r) among the PAHs compounds analysed in PM_2.5_-bound samples of Hulu Langat. (DOCX)

S8 Table Pearson’s correlation between PM_2.5_-bound PAHs and meteorological conditions during the sampling period at the Kuala Lumpur area. (DOCX)

S9 Table Pearson’s correlation between PM_2.5_-bound PAHs and meteorological conditions during the sampling period at the Hulu Langat area. (DOCX)

S10 Table Pearson’s correlation between LMW-, HMW-, and total PAHs with the number of light, heavy, and total vehicles. (DOCX)

S11 Table Factor loadings of PAHs after PCA varimax rotation at Kuala Lumpur. (DOCX)

S12 Table Mass contributions of each emission source that based on the APCS-MLR technique. (DOCX)

S1 Fig Frequencies of air trajectories were obtained (A) in Kuala Lumpur on the 20th March 2021, (B) in Hulu Langat on the 23th April 2021. (JPEG)

S2 Fig Contribution percentage of PAHs based on their rings number to total PAHs, A: In Kuala Lumpur area, B: In Hulu Langat area. (JPEG)

S3 Fig Percentage of LMW-PAHs and HMW-PAHs, A: In Kuala Lumpur area, B: In Hulu Langat area. (JPEG)

S4 Fig Bivariate plot of DRs in the Kuala Lumpur area. (JPEG)

S5 Fig Bivariate plot of DRs in the Hulu Langat area. (JPEG)

S6 Fig Contribution (%) of each emission source determined by APCS/MLR analysis on total PAHs in Kuala Lumpur. (JPEG)

S7 Fig Correlation plot of modeled PAHs by APCS and measured PAHs using GC-MS. (JPEG)
