# Supplementary material for: Effect of traffic volumes on polycyclic aromatic hydrocarbons of particulate matter: A comparative study from urban and rural areas in Malaysia
Source: PLoS One. 2024 Dec 12;19(12):e0315439. doi: 10.1371/journal.pone.0315439 (PMC11637314; doi:10.1371/journal.pone.0315439)
Supplement: S1 Table — (DOCX) [file pone.0315439.s001.docx]

**S1 Table.** Highest traffic volume areas in Peninsular Malaysia [Adapted from the Ministry of Works, Malaysia, 2022].

| **State** | **District** | **Station No.** | **Route No.** | **Section No.** | **Average 16h TV** | **LOS** |
| --- | --- | --- | --- | --- | --- | --- |
| **Selangor** | **Petaling** | **BR807** | **2** | **-** | **260,288** | **F** |
| Kuala Lumpur | Kuala Lumpur | WR103 | 1 | - | 205,686 | F |
| Johor | Johor Bahru | JR205 | 1 | 11 | 184,136 | F |
| Penang | Pulau Pinang | PR208 | 3113 | - | 125,126 | F |
| Perak | Kinta | AR304 | 1 | 604.2 | 81,402 | F |
| Pahang | Kuantan | CR406 | 3 | 333.7 | 65,019 | F |
| Negri Sembilan | Port Dickson | NR305 | 5 | 6 | 53,157 | F |
| Kedah | Kota Setar | KR101 | 175 | - | 54,311 | D |
| Melaka | Melaka Tengah | MR104 | 19 | 5 | 109,500 | F |
| Terengganu | Kuala Terengganu | TR405 | 3685 | 0.2 | 54,860 | F |

Abbreviation: LOS= Load of service= It is graded from A-F according to lowest to highest traffic volume, 16h TV= 16-hour traffic volume
