# Supplementary material for: Effect of traffic volumes on polycyclic aromatic hydrocarbons of particulate matter: A comparative study from urban and rural areas in Malaysia
Source: PLoS One. 2024 Dec 12;19(12):e0315439. doi: 10.1371/journal.pone.0315439 (PMC11637314; doi:10.1371/journal.pone.0315439)
Supplement: S2 Table — (DOCX) [file pone.0315439.s002.docx]

**S2 Table.** Lowest Traffic Volume Areas in Peninsular Malaysia [Adapted from the Ministry of Works, Malaysia, 2022].

| **State** | **District** | **Station No.** | **Route No.** | **Section No.** | **Average 16H TV** | **LOS** |
| --- | --- | --- | --- | --- | --- | --- |
| **Selangor** | **Hulu Langat** | **BR613** | **B32** | **-** | **775** | **A** |
| Kuala Lumpur | Kuala Lumpur | WR106 | - | - | 122,566 | F |
| Johor | Mersing | JR504 | 50 | 137 | 946 | A |
| Penang | Pulau Pinang | PR111 | P143 | - | 3,202 | A |
| Perak | Kinta | AR314 | A119 | 3.2 | 896 | A |
| Pahang | Raub | CR701 | 55 | - | 894 | A |
| Negri Sembilan | Rembau | NR404 | 5 | - | 1,767 | A |
| Kedah | Langkawi | KR804 | 113 | 14.98 | 3,877 | A |
| Melaka | Jasin | MR201 | M8 | 38.6 | 2,850 | A |
| Terengganu | Kemaman | TR301 | T13 | - | 1,007 | A |
| Kelantan | Jeli | DR603 | 4 | 226.9 | 6,344 | A |

Abbreviation: LOS= Load of service= It is graded from A-F according to lowest to highest traffic volume, 16h TV= 16-hour traffic volume
