# Supplementary material for: Effect of traffic volumes on polycyclic aromatic hydrocarbons of particulate matter: A comparative study from urban and rural areas in Malaysia
Source: PLoS One. 2024 Dec 12;19(12):e0315439. doi: 10.1371/journal.pone.0315439 (PMC11637314; doi:10.1371/journal.pone.0315439)
Supplement: S3 Table — (DOCX) [file pone.0315439.s003.docx]

**S3 Table.** Meteorological conditions during air sampling at Kuala Lumpur in 2021.

| **Sample No.** | **Date** | **Temperature**  **°C** | **Humidity**  **(%)** | **Wind Speed**  **(km h^-1^)** | **Wind Direction** |
| --- | --- | --- | --- | --- | --- |
| 1 | 09/03 | 35 | 44 | 17 | W |
| 2 | 10/03 | 34 | 56 | 15 | NW |
| 3 | 11/03 | 34 | 53 | 9 | NW |
| 4 | 12/03 | 33 | 59 | 15 | NW |
| 5 | 13/03 | 29 | 74 | 6 | SW |
| 6 | 14/03 | 32 | 67 | 11 | WSW |
| 7 | 15/03 | 34 | 53 | 7 | NWbW |
| 8 | 16/03 | 34 | 53 | 15 | NWbN |
| 9 | 17/03 | 33 | 63 | 20 | WSW |
| 10 | 18/03 | 33 | 63 | 2 | WbN |
| 11 | 19/03 | 26 | 89 | 31 | NW |
| 12 | 20/03 | 33 | 63 | 17 | WbS |
| 13 | 21/03 | 30 | 75 | 17 | SW |
| 14 | 23/03 | 30 | 70 | 13 | WbN |
| 15 | 24/03 | 30 | 70 | 6 | N |
| 16 | 25/03 | 33 | 52 | 7 | NW |
| 17 | 26/03 | 32 | 55 | 7 | N |
| 18 | 27/03 | 33 | 56 | 4 | WSW |
| 19 | 28/03 | 31 | 62 | 7 | NWbW |
| 20 | 31/03 | 35 | 50 | 15 | NW |

Abbreviation: N: North NW: North-west NWbN: Northwest by north SW: South-west W: West WbN: West by north WbS: West by south WSW: West-southwest
