# Supplementary material for: Effect of traffic volumes on polycyclic aromatic hydrocarbons of particulate matter: A comparative study from urban and rural areas in Malaysia
Source: PLoS One. 2024 Dec 12;19(12):e0315439. doi: 10.1371/journal.pone.0315439 (PMC11637314; doi:10.1371/journal.pone.0315439)
Supplement: S4 Table — (DOCX) [file pone.0315439.s004.docx]

**S4 Table.** Meteorological conditions during air sampling at Hulu Langat in 2021.

| **Sample No.** | **Date** | **Temperature**  **°C** | **Humidity**  **(%)** | **Wind Speed**  **(km h^-1^)** | **Wind Direction** |
| --- | --- | --- | --- | --- | --- |
| 1 | 11/04 | 32 | 74 | 7 | NWbN |
| 2 | 12/04 | 31 | 73 | 5 | NW |
| 3 | 13/04 | 30 | 73 | 4 | WSW |
| 4 | 14/04 | 29 | 70 | 4 | SW |
| 5 | 15/04 | 30 | 70 | 7 | NNW |
| 6 | 16/04 | 29 | 70 | 4 | WSW |
| 7 | 17/04 | 30 | 70 | 2 | NWbN |
| 8 | 18/04 | 30 | 75 | 4 | N |
| 9 | 19/04 | 28 | 70 | 11 | NNW |
| 10 | 20/04 | 28 | 74 | 11 | NWbN |
| 11 | 21/04 | 30 | 75 | 4 | WbN |
| 12 | 22/04 | 29 | 74 | 4 | NW |
| 13 | 23/04 | 28 | 84 | 4 | WbS |
| 14 | 24/04 | 29 | 74 | 4 | SW |
| 15 | 25/04 | 28 | 79 | 4 | NNW |
| 16 | 26/04 | 28 | 79 | 7 | SbW |
| 17 | 27/04 | 29 | 74 | 6 | W |
| 18 | 28/04 | 29 | 74 | 13 | SbW |
| 19 | 29/04 | 30 | 75 | 2 | NWbW |
| 20 | 30/04 | 27 | 79 | 4 | WbN |

Abbreviation: N: North NNW: North-northwest NW: North-west NWbN: Northwest by north SbW: South by west SW: South-west W: West WbN: West by north WbS: West by south WSW: West-southwest
