# Supplementary material for: Effect of traffic volumes on polycyclic aromatic hydrocarbons of particulate matter: A comparative study from urban and rural areas in Malaysia
Source: PLoS One. 2024 Dec 12;19(12):e0315439. doi: 10.1371/journal.pone.0315439 (PMC11637314; doi:10.1371/journal.pone.0315439)
Supplement: S5 Table — (DOCX) [file pone.0315439.s005.docx]

**S5 Table.** LOD and Matrix-Standard Spiking Recovery of 16 PAHs.

|  | **PAH**  **Compound** | **LOD**  **(ppb)** | **Matrix-Standard Spiking Recovery**  **(%)** |
| --- | --- | --- | --- |
|  | NAP | 0.06 | 76.8±7.3 |
|  | ACE | 0.01 | 88.8±8.9 |
|  | ACY | 0.04 | 95.4±14.3 |
|  | FLO | 0.04 | 101.3±10 |
|  | PHE | 0.03 | 98±9.4 |
|  | ANT | 0.08 | 103.3±13.1 |
|  | FLA | 0.06 | 97.6±16.4 |
|  | PYR | 0.06 | 99.5±11.3 |
|  | BAA | 0.06 | 58±15.1 |
|  | CHR | 0.08 | 64±16.1 |
|  | BKF | 0.06 | 48±6.53 |
|  | BAP | 0.02 | 53.4±7.6 |
|  | BBF | 0.05 | 53±7.22 |
|  | IcdP | 0.05 | 81±8.65 |
|  | DahA | 0.02 | 60.5±7.13 |
|  | BghiP | 0.02 | 60.1±5.75 |

Abbreviations: LOD= Limit of detection ppb= Parts per billion
