# Supplementary material for: Effect of traffic volumes on polycyclic aromatic hydrocarbons of particulate matter: A comparative study from urban and rural areas in Malaysia
Source: PLoS One. 2024 Dec 12;19(12):e0315439. doi: 10.1371/journal.pone.0315439 (PMC11637314; doi:10.1371/journal.pone.0315439)
Supplement: S6 Table — (DOCX) [file pone.0315439.s006.docx]

**S6 Table.** Pearson correlation coefficients (r) among the PAHs compounds analysed in PM_2.5_-bound samples of Kuala Lumpur.

|  | NAP | ACY | ACP | FLR | ANT | PHE | FLT | PYR | BaA | CHR | BkF | BaP | BbF | IcP | DhA | BgP |
| --- | --- | --- | --- | --- | --- | --- | --- | --- | --- | --- | --- | --- | --- | --- | --- | --- |
| NAP | 1 | 0.33 | 0.62** | 0.62** | 0.49 | 0.76** | 0.89** | 0.64** | 0.44 | 0.68** | 0.17 | 0.45* | 0.28 | 0.30 | 0.47 | 0.50 |
| ACY |  | 1 | 0.30 | 0.62** | 0.39 | 0.56 ** | 0.31 | 0.53 | 0.47 | 0.37 | 0.26 | 0.18 | 0.28 | 0.43 | 0.43 | 0.30 |
| ACP |  |  | 1 | 0.51 | 0.48 | 0.56 | 0.65** | 0.40 | 0.34 | 0.56 | 0.23 | 0.05 | 0.40 | 0.56 | 0.46 | 0.33 |
| FLR |  |  |  | 1 | 0.55 | 0.72** | 0.06** | 0.69** | 0.48 | 0.65** | 0.40 | 0.180 | 0.30 | 0.45 | 0.48 | 0.35 |
| PHE |  |  |  |  | 1 | 0.76** | 0.47 | 0.57** | 0.44 | 0.50 | 0.27 | 0.40 | 0.14 | 0.01 | 0.09 | 0.19 |
| ANT |  |  |  |  |  | 1 | 0.76** | 0.76** | 0.55 | 0.57 | 0.21 | 0.40 | 0.34 | 0.22 | 0.47 | 0.45 |
| FLT |  |  |  |  |  |  | 1 | 0.66** | 0.52 | 0.61** | 0.35 | 0.25 | 0.46 | 0.43 | 0.26 | 0.81** |
| PYR |  |  |  |  |  |  |  | 1 | 0.51 | 0.45 | 0.13 | 0.34 | 0.28 | 0.35 | 0.56 | 0.55 |
| BaA |  |  |  |  |  |  |  |  | 1 | 0.71** | 0.12 | 0.63** | 0.67** | 0.26 | 0.36 | 0.71** |
| CHR |  |  |  |  |  |  |  |  |  | 1 | 0.17 | 0.09 | 0.83** | 0.40 | 0.65** | 0.46 |
| BkF |  |  |  |  |  |  |  |  |  |  | 1 | 0.73** | 0.22 | 0.21 | 0.66** | 0.38 |
| BaP |  |  |  |  |  |  |  |  |  |  |  | 1 | 0.20 | 0.19 | 0.62** | 0.22 |
| BbF |  |  |  |  |  |  |  |  |  |  |  |  | 1 | 0.74** | 0.80** | 0.32 |
| IcP |  |  |  |  |  |  |  |  |  |  |  |  |  | 1 | 0.84** | 0.60** |
| DhA |  |  |  |  |  |  |  |  |  |  |  |  |  |  | 1 | 0.50 |
| BgP |  |  |  |  |  |  |  |  |  |  |  |  |  |  |  | 1 |

Abbreviation: *: significant p = <0.05 **: significant p = <0.01
