# Supplementary material for: Effect of traffic volumes on polycyclic aromatic hydrocarbons of particulate matter: A comparative study from urban and rural areas in Malaysia
Source: PLoS One. 2024 Dec 12;19(12):e0315439. doi: 10.1371/journal.pone.0315439 (PMC11637314; doi:10.1371/journal.pone.0315439)
Supplement: S7 Table — (DOCX) [file pone.0315439.s007.docx]

**S7 Table.** Pearson correlation coefficients (r) among the PAHs compounds analysed in PM_2.5_-bound samples of Hulu Langat.

|  | NAP | ACY | ACP | FLR | ANT | PHE | FLT | PYR | BaA | CHR | BkF | BaP | BbF | IcP | DhA | BgP |
| --- | --- | --- | --- | --- | --- | --- | --- | --- | --- | --- | --- | --- | --- | --- | --- | --- |
| NAP | 1 | 0.68** | 0.22 | 0.06 | 0.39 | 0.52 | 0.55 | 0.35 | 0.39 | 0.24 | 0.09 | 0.12 | 0.22 | 0.01 | 0.18 | 0.11 |
| ACY |  | 1 | 0.33 | 0.16 | 0.60** | 0.64** | 0.68** | 0.59** | 0.52 | 0.32 | 0.4 | 0.37 | 0.34 | 0.23 | 0.22 | 0.03 |
| ACP |  |  | 1 | 0.01 | 0.14 | 0.25 | 0.39 | 0.22 | 0.23 | 0.1 | 0.14 | 0.21 | 0.08 | 0.21 | 0.5 | 0.11 |
| FLR |  |  |  | 1 | 0.22 | 0.19 | 0.14 | 0.45 | 0.34 | 0.25 | 0.12 | 0.16 | 0.2 | 0.25 | 0.29 | 0.23 |
| PHE |  |  |  |  | 1 | 0.91** | 0.71** | 0.56** | 0.61** | 0.36 | 0.35 | 0.05 | 0.44 | 0.16 | 0.07 | 0.14 |
| ANT |  |  |  |  |  | 1 | 0.75** | 0.64** | 0.60** | 0.42 | 0.36 | 0.13 | 0.45 | 0.21 | 0.22 | 0.17 |
| FLT |  |  |  |  |  |  | 1 | 0.8** | 0.76** | 0.69** | 0.33 | 0.01 | 0.56** | 0.14 | 0.23 | 0.15 |
| PYR |  |  |  |  |  |  |  | 1 | 0.5 | 0.71** | 0.44 | 0.04 | 0.33 | 0.1 | 0.05 | 0.1 |
| BaA |  |  |  |  |  |  |  |  | 1 | 0.59** | 0.27 | 0.36 | 0.82** | 0.68** | 0.44 | 0.63** |
| CHR |  |  |  |  |  |  |  |  |  | 1 | 0.47 | 0.28 | 0.77** | 0.11 | 0.29 | 0.88** |
| BkF |  |  |  |  |  |  |  |  |  |  | 1 | 0.19 | 0.17 | 0.32 | 0.66** | 0.42 |
| BaP |  |  |  |  |  |  |  |  |  |  |  | 1 | 0.11 | 0.16 | 0.79** | 0.17 |
| BbF |  |  |  |  |  |  |  |  |  |  |  |  | 1 | 0.2 | 0.49 | 0.75** |
| IcP |  |  |  |  |  |  |  |  |  |  |  |  |  | 1 | 0.69** | 0.70** |
| DhA |  |  |  |  |  |  |  |  |  |  |  |  |  |  | 1 | 0.66** |
| BgP |  |  |  |  |  |  |  |  |  |  |  |  |  |  |  | 1 |

Abbreviation: *: significant p = <0.05 **: significant p = <0.01
