# Supplementary material for: Effect of traffic volumes on polycyclic aromatic hydrocarbons of particulate matter: A comparative study from urban and rural areas in Malaysia
Source: PLoS One. 2024 Dec 12;19(12):e0315439. doi: 10.1371/journal.pone.0315439 (PMC11637314; doi:10.1371/journal.pone.0315439)
Supplement: S8 Table — (DOCX) [file pone.0315439.s008.docx]

**S8 Table.** Pearson’s correlation between PM_2.5_-bound PAHs and meteorological conditions during the sampling period at the Kuala Lumpur area.

|  | Temperature | Humidity | Wind Speed | Air Pressure |
| --- | --- | --- | --- | --- |
| Temperature | 1 | -0.93** | -0.26 | 0.33 |
| Humidity | -0.93** | 1 | 0.35 | 0.12 |
| Wind Speed | -0.26 | 0.35 | 1 | -0.37 |
| Air Pressure | -0.33 | 0.12 | 0.12 | 1 |
| NAP | -0.24 | 0.10 | 0.05 | -0.38 |
| ACY | -0.22 | 0.34 | 0.28 | 0.36 |
| ACP | -0.07 | 0.14 | 0.51* | -0.02 |
| FLR | -0.41 | 0.46* | 0.15 | 0.12 |
| PHE | -0.22 | 0.37 | 0.21 | 0.21 |
| ANT | -0.28 | 0.38 | 0.14 | 0.01 |
| FLT | -0.25 | 0.22 | 0.11 | -0.01 |
| PYR | -0.26 | 0.22 | 0.03 | -0.31 |
| BaA | -0.14 | 0.12 | 0.14 | 0.19 |
| CHR | -0.19 | 0.13 | 0.01 | 0.09 |
| BkF | -0.15 | 0.06 | 0.22 | -0.44 |
| BaP | -0.36 | 0.32 | 0.05 | 0.02 |
| BbF | -0.04 | 0.05 | 0.20 | 0.07 |
| IcP | -0.34 | 0.36 | 0.06 | 0.06 |
| DhA | -0.26 | 0.13 | 0.17 | -0.40 |
| BgP | -0.47* | 0.40 | 0.16 | -0.35 |

Abbreviation: *: significant p = <0.05 **: significant p = <0.01
