# Supplementary material for: Effect of traffic volumes on polycyclic aromatic hydrocarbons of particulate matter: A comparative study from urban and rural areas in Malaysia
Source: PLoS One. 2024 Dec 12;19(12):e0315439. doi: 10.1371/journal.pone.0315439 (PMC11637314; doi:10.1371/journal.pone.0315439)
Supplement: S9 Table — (DOCX) [file pone.0315439.s009.docx]

**S9 Table.** Pearson’s correlation between PM_2.5_-bound PAHs and meteorological conditions during the sampling period at the Hulu Langat area.

|  | Temperature | Humidity | Wind Speed | Air Pressure |
| --- | --- | --- | --- | --- |
| Temperature | 1 | -0.67** | -0.17 | 0.11 |
| Humidity | -0.67** | 1 | -0.16 | 0.19 |
| Wind Speed | -0.17 | -0.16 | 1 | -0.01 |
| Air Pressure | -0.11 | 0.19 | -0.01 | 1 |
| NAP | -0.39 | 0.12 | 0.24 | -0.37 |
| ACY | -0.39 | 0.12 | 0.24 | -0.37 |
| ACP | -0.39 | 0.12 | 0.24 | -0.37 |
| FLR | -0.39 | 0.12 | 0.24 | -0.37 |
| PHE | -0.39 | 0.12 | 0.24 | -0.37 |
| ANT | -0.39 | 0.12 | 0.24 | -0.37 |
| FLT | -0.39 | 0.12 | 0.24 | -0.37 |
| PYR | -.471* | 0.31 | 0.21 | -0.37 |
| BaA | -0.39 | 0.12 | 0.24 | -0.37 |
| CHR | -0.39 | 0.12 | 0.24 | -0.37 |
| BkF | -0.39 | 0.12 | 0.24 | -0.37 |
| BaP | -0.39 | 0.12 | 0.24 | -0.37 |
| BbF | -0.39 | 0.12 | 0.24 | -0.37 |
| IcP | -0.39 | 0.12 | 0.24 | -0.37 |
| DhA | -0.39 | 0.12 | 0.24 | -0.37 |
| BgP | -0.39 | 0.12 | 0.24 | -0.37 |

Abbreviation: *: significant p = <0.05 **: significant p = <0.01
