# Supplementary material for: Effect of traffic volumes on polycyclic aromatic hydrocarbons of particulate matter: A comparative study from urban and rural areas in Malaysia
Source: PLoS One. 2024 Dec 12;19(12):e0315439. doi: 10.1371/journal.pone.0315439 (PMC11637314; doi:10.1371/journal.pone.0315439)
Supplement: S10 Table — (DOCX) [file pone.0315439.s010.docx]

**S10 Table.** Pearson’s correlation between LMW-, HMW-, and total PAHs with the number of light, heavy, and total vehicles.

|  | Light Vehicles | Heavy Vehicles | Total Vehicles |
| --- | --- | --- | --- |
| LMW-PAHs | 0.79^**^ | 0.85^**^ | 0.76^**^ |
| HMW-PAHs | 0.86^**^ | 0.78^**^ | 0.87^**^ |
| Total PAHs | 0.84^**^ | 0.81^**^ | 0.83^**^ |

Abbreviation: *: significant p = <0.05 **: significant p = <0.01
