# Supplementary material for: Effect of traffic volumes on polycyclic aromatic hydrocarbons of particulate matter: A comparative study from urban and rural areas in Malaysia
Source: PLoS One. 2024 Dec 12;19(12):e0315439. doi: 10.1371/journal.pone.0315439 (PMC11637314; doi:10.1371/journal.pone.0315439)
Supplement: S11 Table — (DOCX) [file pone.0315439.s011.docx]

**S11 Table.** Factor loadings of PAHs after PCA varimax rotation at Kuala Lumpur.

|  | **Rotated Component Matrix** | | |
| --- | --- | --- | --- |
|  | **Factor 1** | **Factor 2** | **Factor 3** |
| NAP | **0.608** | -0.494 | -0.322 |
| ACP | 0.425 | 0.362 | -0.070 |
| ACY | -0.319 | -0.180 | 0.017 |
| FLR | 0.314 | 0.290 | 0.409 |
| PHE | 0.116 | **0.864** | -0.154 |
| ANT | -0.084 | **0.904** | 0.146 |
| FLT | **0.664** | -0.100 | -0.042 |
| PYR | **0.641** | 0.062 | -0.064 |
| BaA | **0.664** | -0.042 | -0.129 |
| CHR | **0.625** | 0.036 | 0.152 |
| BkF | 0.041 | -0.489 | **0.728** |
| BaP | -0.574 | 0.278 | **0.747** |
| BbF | -0.492 | **0.677** | 0.198 |
| IcP | -0.026 | **0.735** | 0.066 |
| DhA | 0.275 | **0.811** | -0.054 |
| BgP | 0.156 | **0.645** | 0.055 |
| Eigen value | 2.86 | 2.56 | 2.38 |
| % of Variance | 22.02 | 20.85 | 19.78 |
| Cumulative % | 22.02 | 42.87 | 62.65 |

= <0.01
