# Supplementary material for: Effect of traffic volumes on polycyclic aromatic hydrocarbons of particulate matter: A comparative study from urban and rural areas in Malaysia
Source: PLoS One. 2024 Dec 12;19(12):e0315439. doi: 10.1371/journal.pone.0315439 (PMC11637314; doi:10.1371/journal.pone.0315439)
Supplement: S12 Table — (DOCX) [file pone.0315439.s012.docx]

**S12 Table****.** Mass contributions of each emission source that based on the APCS-MLR technique.

| **PAH** | **Unit** | **Factor1** | **Factor 2** | **Factor 3** | **Unaccounted** | **Modeled** | **Measured** | **R^2^** | **Error (%)** |
| --- | --- | --- | --- | --- | --- | --- | --- | --- | --- |
| **NAP** | ng m^-3^ | 0.45 | 0 | 0 | 0 | 0.45 | 0.44 | 0.96 | 2.27 |
| **ACE** | ng m^-3^ | 0 | 0.27 | 0 | 0 | 0.27 | 0.25 | 0.97 | 8.00 |
| **ACY** | ng m^-3^ | 0 | 0 | 0.18 | 0.06 | 0.24 | 0.23 | 0.96 | 4.35 |
| **FLR** | ng m^-3^ | 0 | 0.1 | 0.22 | 0.07 | 0.39 | 0.37 | 0.98 | 5.41 |
| **PHE** | ng m^-3^ | 0 | 0.31 | 0 | 0.04 | 0.35 | 0.34 | 0.96 | 2.94 |
| **ANT** | ng m^-3^ | 0 | 0.23 | 0 | 0.13 | 0.36 | 0.33 | 0.97 | 9.09 |
| **FLT** | ng m^-3^ | 0.35 | 0 | 0 | 0 | 0.35 | 0.34 | 0.94 | 2.94 |
| **PYR** | ng m^-3^ | 0.32 | 0 | 0 | 0 | 0.32 | 0.32 | 0.92 | 0.00 |
| **BaA** | ng m^-3^ | 0.29 | 0 | 0 | 0.06 | 0.35 | 0.35 | - | 0.00 |
| **CHY** | ng m^-3^ | 0.48 | 0.04 | 0.05 | 0.07 | 0.64 | 0.63 | 0.93 | 1.59 |
| **BkF** | ng m^-3^ | 0 | 0 | 0.33 | 0 | 0.33 | 0.33 | 0.93 | 0.00 |
| **BaP** | ng m^-3^ | 0 | 0 | 0.46 | 0 | 0.46 | 0.45 | 0.97 | 2.22 |
| **BbF** | ng m^-3^ | 0 | 0.62 | 0.06 | 0 | 0.68 | 0.67 | 0.97 | 1.49 |
| **IcP** | ng m^-3^ | 0 | 0.48 | 0 | 0 | 0.48 | 0.47 | 0.96 | 2.13 |
| **DhA** | ng m^-3^ | 0 | 0.19 | 0 | 0 | 0.19 | 0.17 | 0.96 | 11.76 |
| **BgP** | ng m^-3^ | 0 | 0.14 | 0 | 0 | 0.14 | 0.14 | 0.97 | 0.00 |
| **Total PAHs** | ng m^-3^ | 1.89 | 2.38 | 1.30 | 0.43 | 6 | 5.85 | 0.99 | 2.56 |
