# Supplementary figures and images for: Effect of traffic volumes on polycyclic aromatic hydrocarbons of particulate matter: A comparative study from urban and rural areas in Malaysia
Source: PLoS One. 2024 Dec 12;19(12):e0315439. doi: 10.1371/journal.pone.0315439 (PMC11637314; doi:10.1371/journal.pone.0315439)

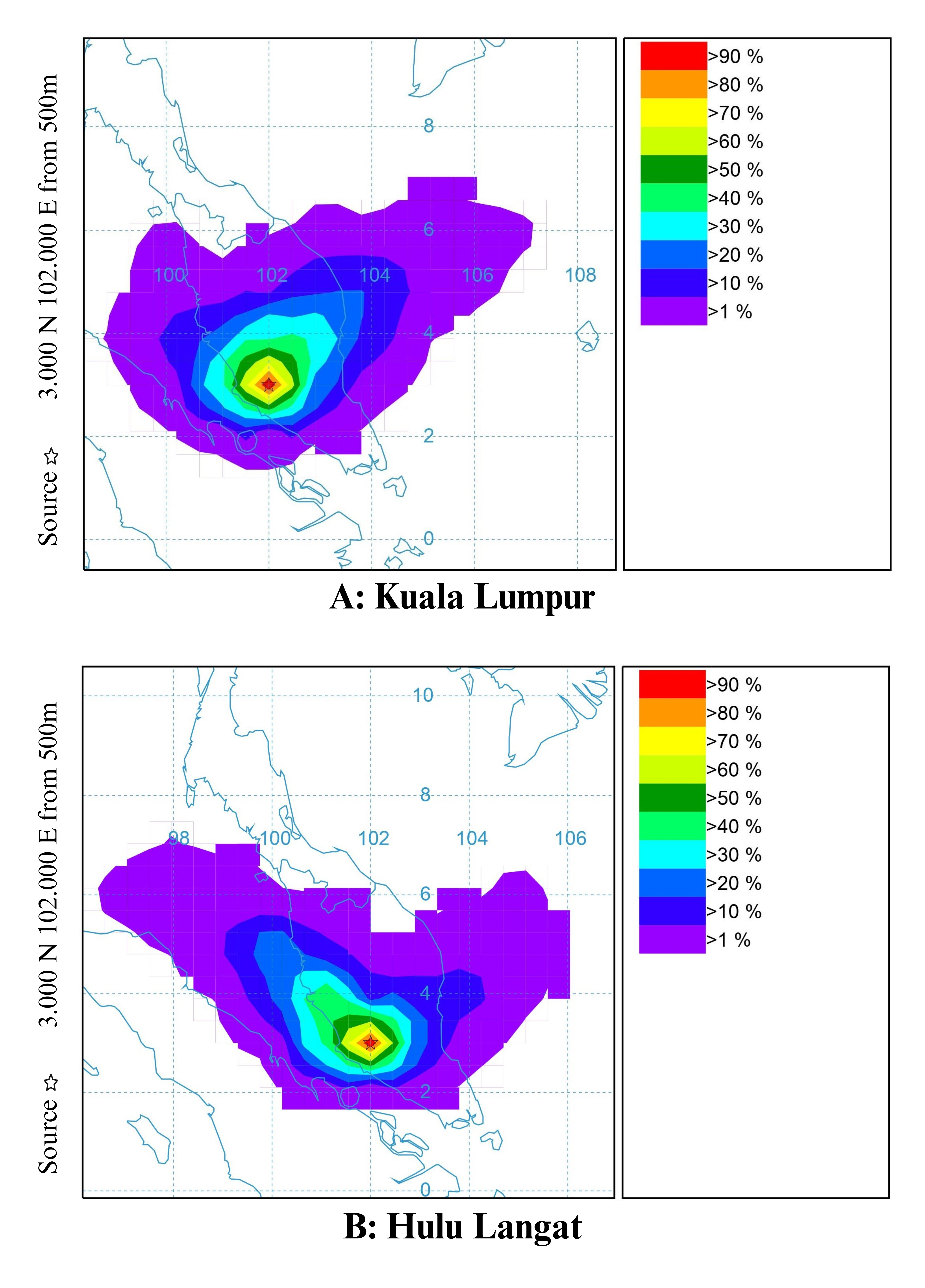

Supplement: S1 Fig — (JPEG) [file pone.0315439.s013.jpeg]

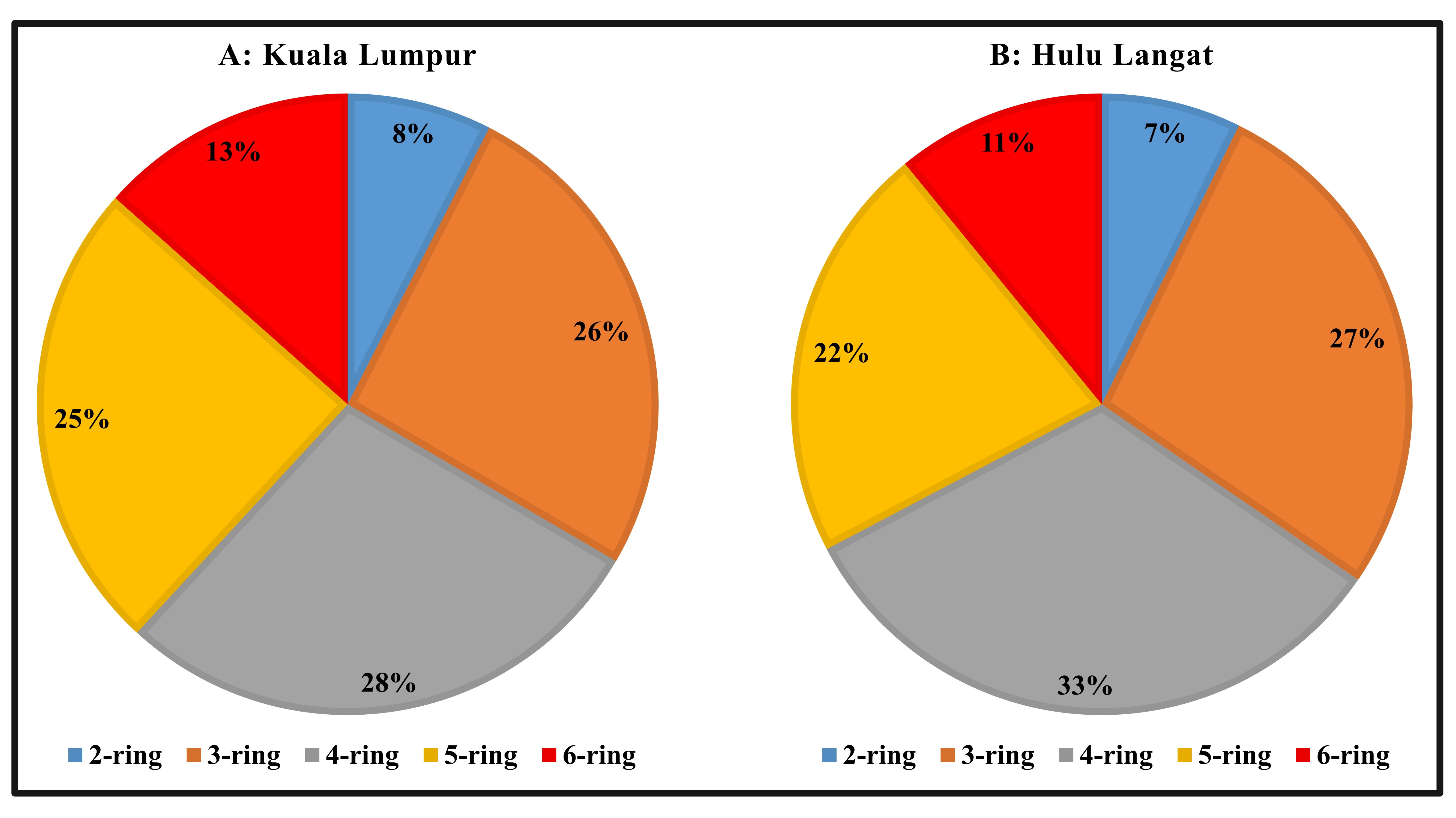

Supplement: S2 Fig — (JPEG) [file pone.0315439.s014.jpeg]

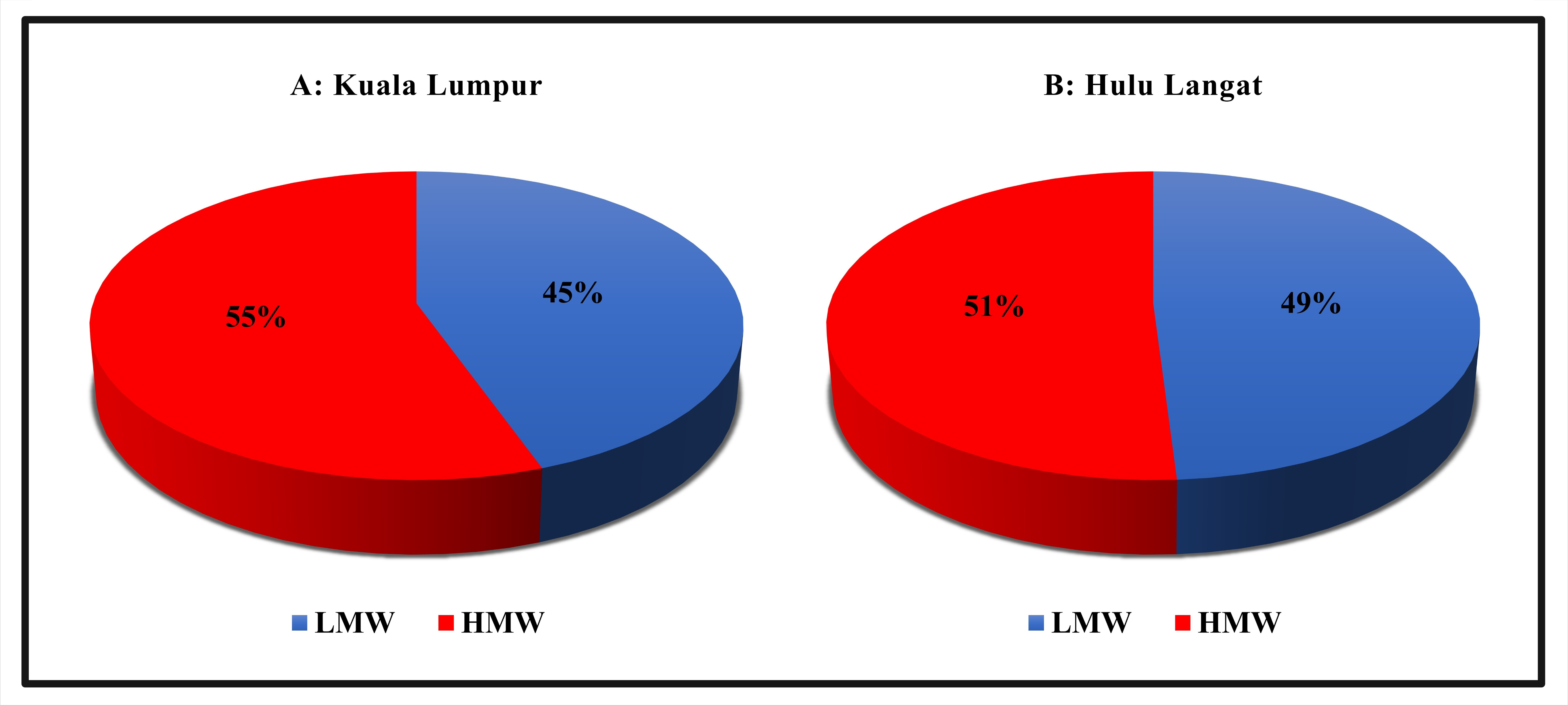

Supplement: S3 Fig — (JPEG) [file pone.0315439.s015.jpeg]

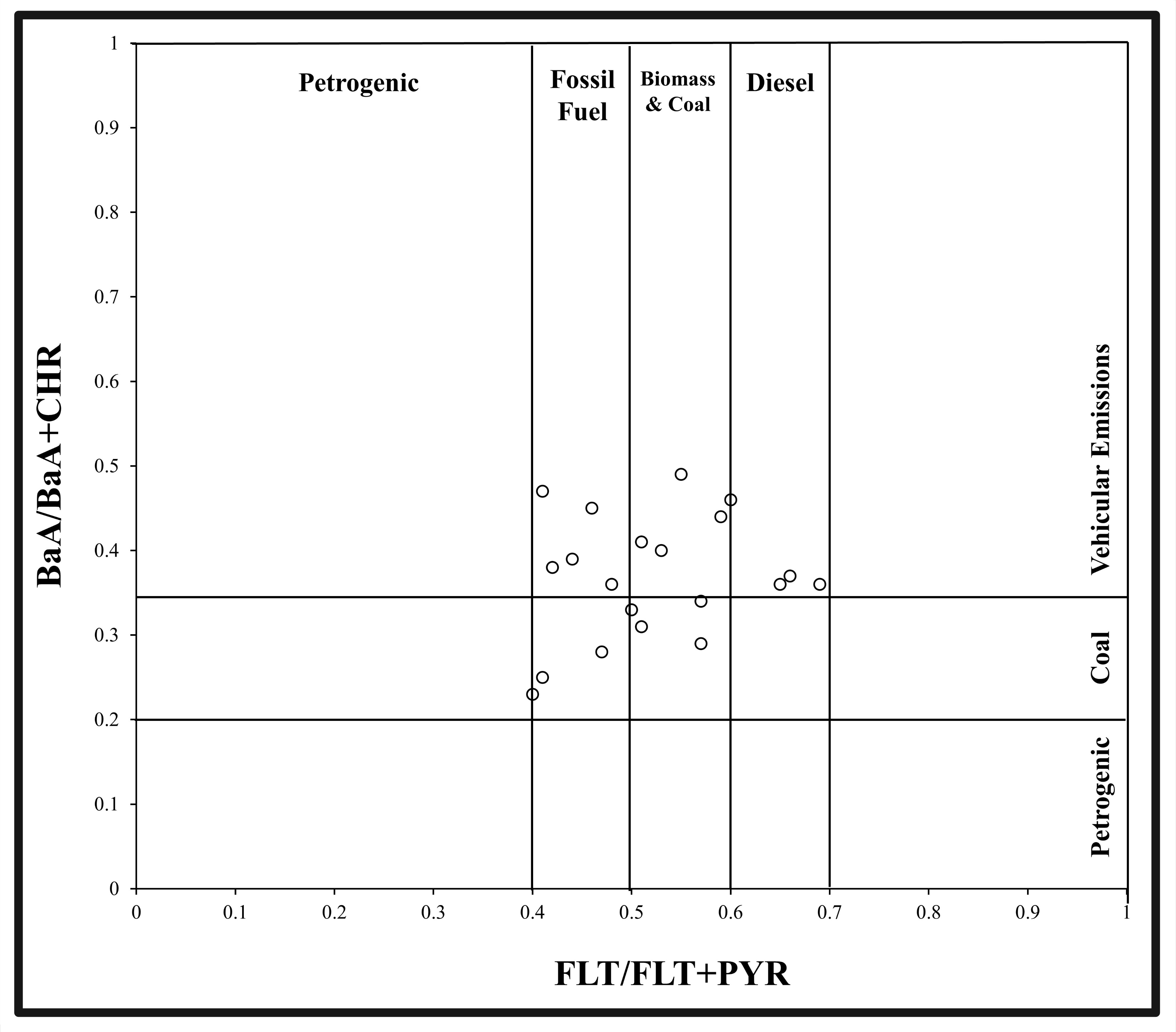

Supplement: S4 Fig — (JPEG) [file pone.0315439.s016.jpeg]

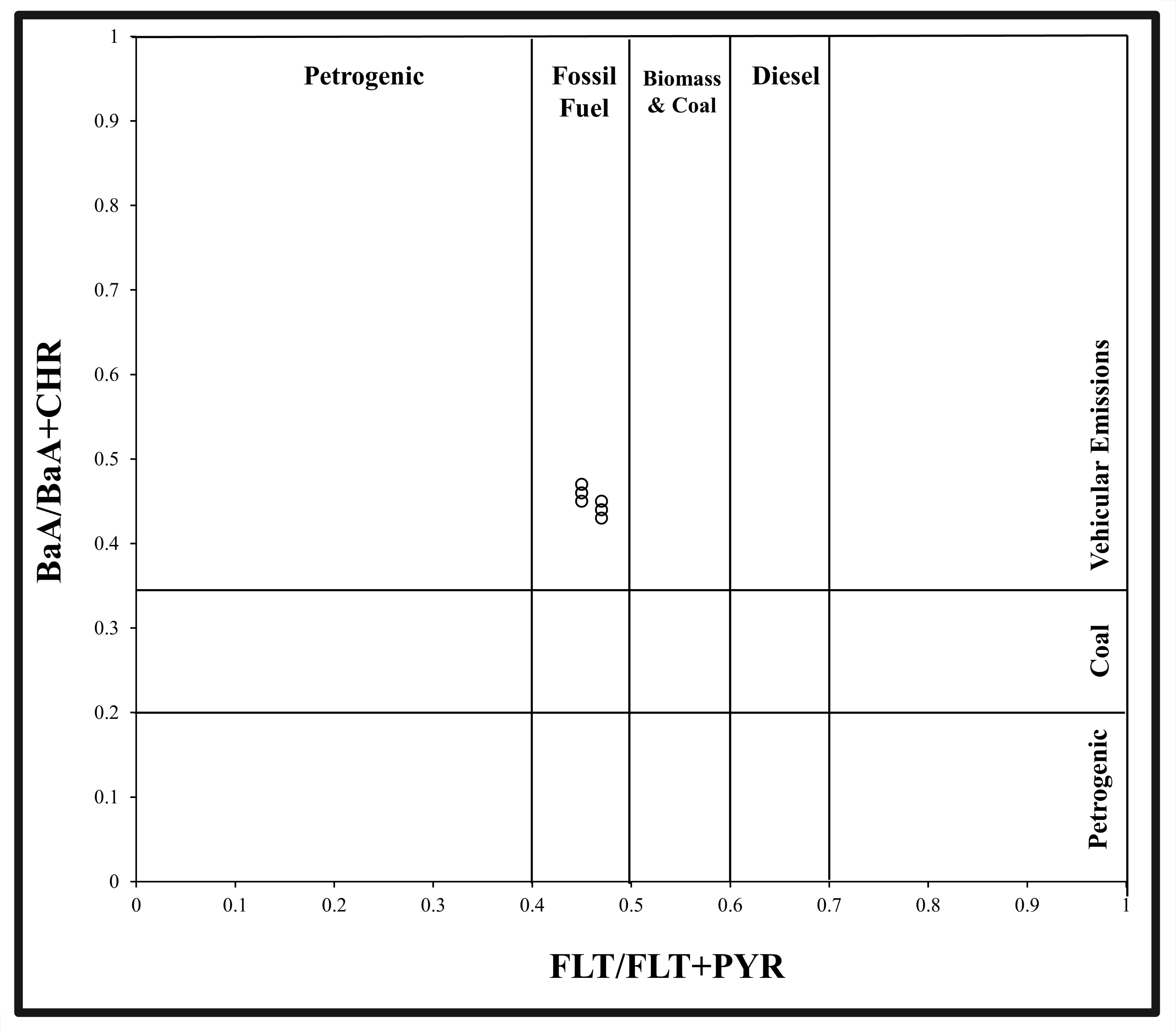

Supplement: S5 Fig — (JPEG) [file pone.0315439.s017.jpeg]

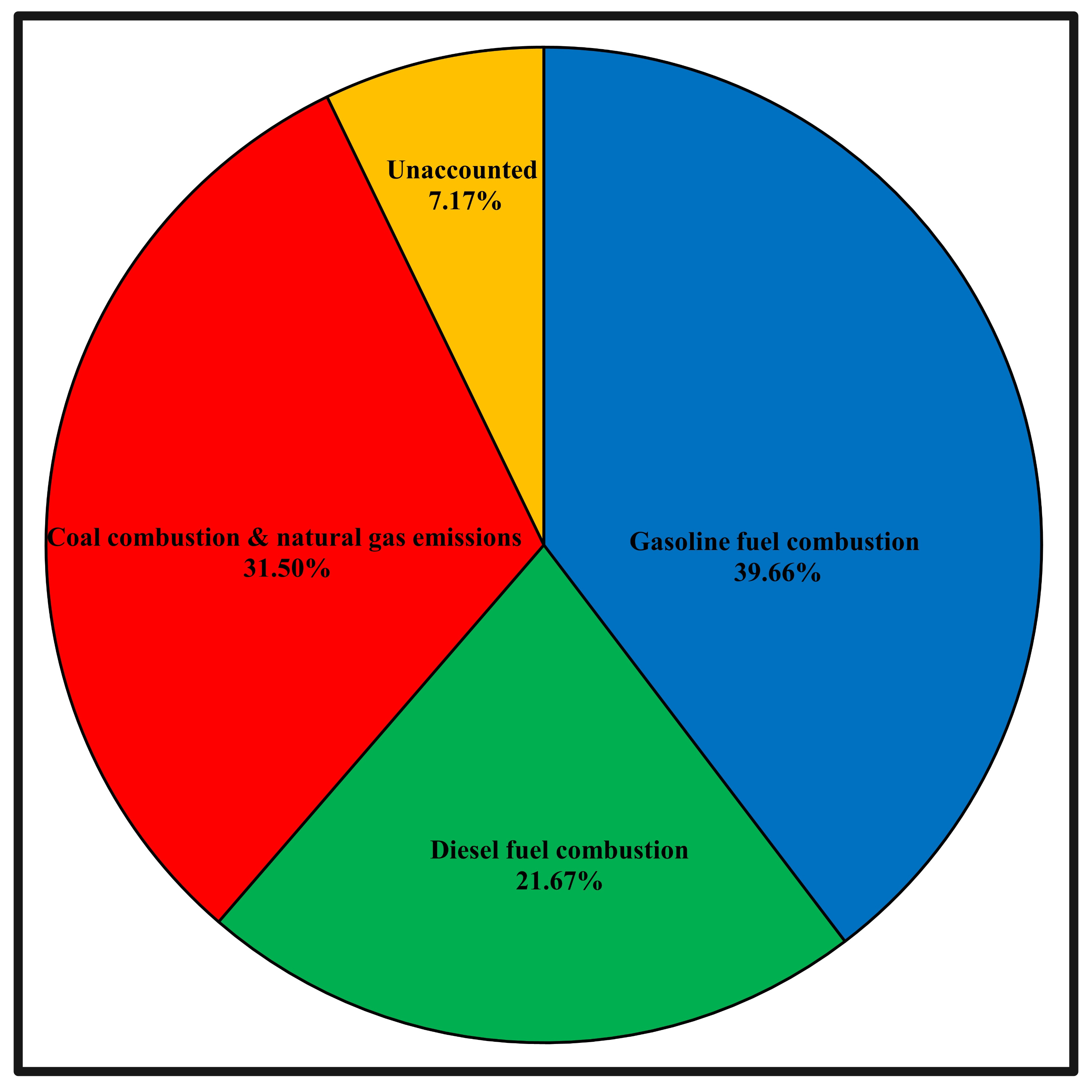

Supplement: S6 Fig — (JPEG) [file pone.0315439.s018.jpeg]

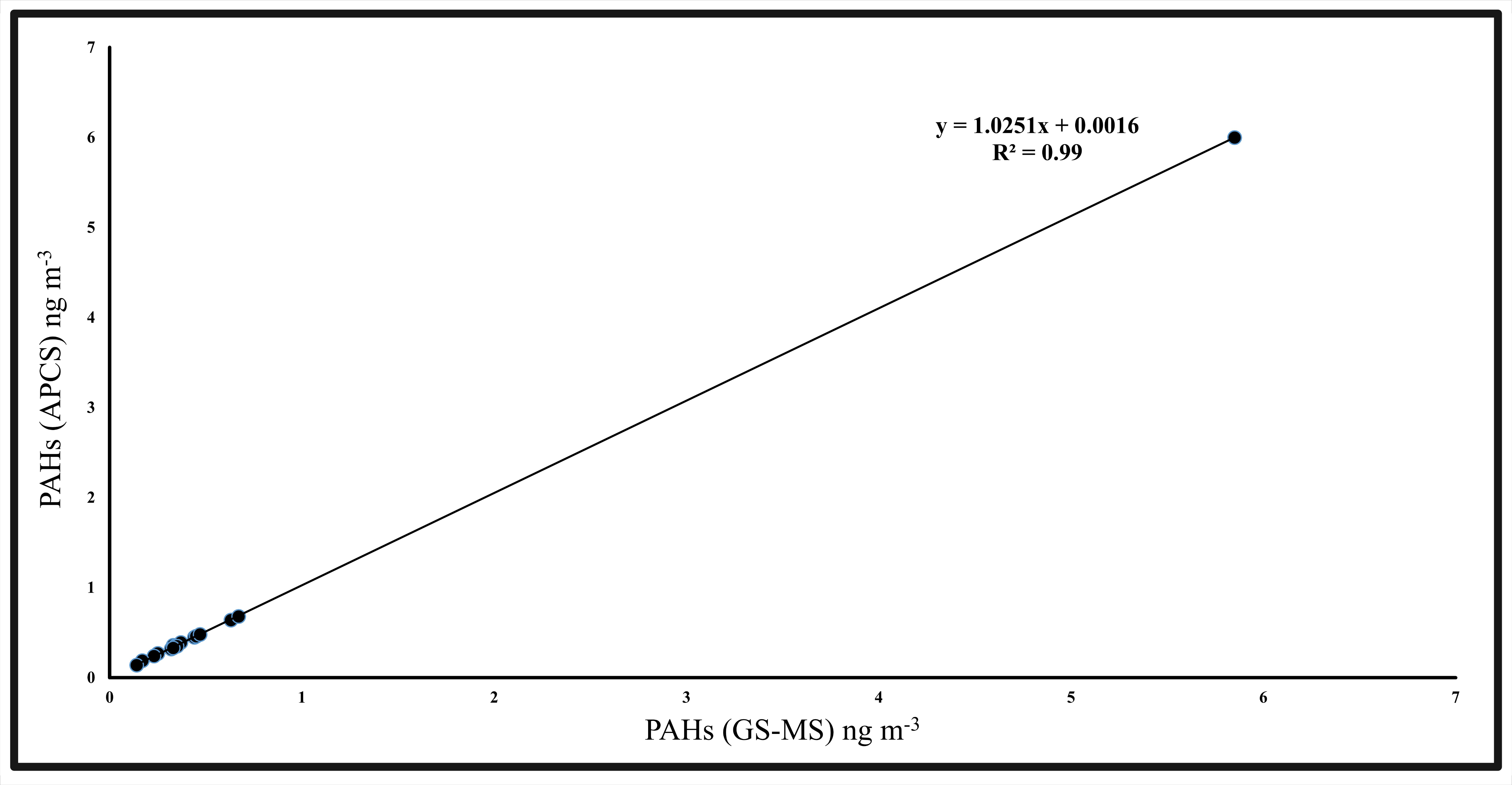

Supplement: S7 Fig — (JPEG) [file pone.0315439.s019.jpeg]
